# Increased Medial Temporal Tau Positron Emission Tomography Uptake in the Absence of Amyloid-β Positivity

**DOI:** 10.1001/jamaneurol.2023.2560

**Published:** 2023-08-14

**Authors:** Alejandro Costoya-Sánchez, Alexis Moscoso, Jesús Silva-Rodríguez, Michael J. Pontecorvo, Michael D. Devous, Pablo Aguiar, Michael Schöll, Michel J. Grothe

**Affiliations:** 1 Universidade de Santiago de Compostela, Santiago de Compostela, Spain; 2 Nuclear Medicine Department and Molecular Imaging Group, Instituto de Investigación Sanitaria de Santiago de Compostel, Travesía da Choupana s/n, Santiago de Compostela, Spain; 3 Centro de Investigación Biomédica en Red sobre Enfermedades Neurodegenerativas, Instituto de Salud Carlos III, Madrid, Spain; 4 Wallenberg Centre for Molecular and Translational Medicine, University of Gothenburg, Gothenburg, Sweden; 5 Department of Psychiatry and Neurochemistry, Institute of Physiology and Neuroscience, University of Gothenburg, Gothenburg, Sweden; 6 Unidad de Trastornos del Movimiento, Servicio de Neurología y Neurofisiología Clínica, Instituto de Biomedicina de Sevilla, Hospital Universitario Virgen del Rocío/CSIC/Universidad de Sevilla, Seville, Spain; 7 Avid Radiopharmaceuticals, Philadelphia, Pennsylvania; 8 Eli Lilly and Company, Indianapolis, Indiana; 9 Dementia Research Centre, Institute of Neurology, University College London, London, United Kingdom

## Abstract

**Importance:**

An increased tau positron emission tomography (PET) signal in the medial temporal lobe (MTL) has been observed in older individuals in the absence of amyloid-β (Aβ) pathology. Little is known about the longitudinal course of this condition, and its association with Alzheimer disease (AD) remains unclear.

**Objective:**

To study the pathologic and clinical course of older individuals with PET-evidenced MTL tau deposition (TMTL^+^) in the absence of Aβ pathology (A^−^), and the association of this condition with the AD continuum.

**Design, Setting, and Participants:**

A multicentric, observational, longitudinal cohort study was conducted using pooled data from the Alzheimer’s Disease Neuroimaging Initiative (ADNI), Harvard Aging Brain Study (HABS), and the AVID-A05 study, collected between July 2, 2015, and August 23, 2021. Participants in the ADNI, HABS, and AVID-A05 studies (N = 1093) with varying degrees of cognitive performance were deemed eligible if they had available tau PET, Aβ PET, and magnetic resonance imaging scans at baseline. Of these, 128 participants did not meet inclusion criteria based on Aβ PET and tau PET biomarker profiles (A^+^ TMTL^−^).

**Exposures:**

Tau and Aβ PET, magnetic resonance imaging, cerebrospinal fluid biomarkers, and cognitive assessments.

**Main Outcomes and Measures:**

Cross-sectional and longitudinal measures for tau and Aβ PET, cortical atrophy, cognitive scores, and core AD cerebrospinal fluid biomarkers (Aβ42/40 and tau phosphorylated at threonine 181 p-tau181 available in a subset).

**Results:**

Among the 965 individuals included in the study, 503 were women (52.1%) and the mean (SD) age was 73.9 (8.1) years. A total of 51% of A^−^ individuals and 78% of A^+^ participants had increased tau PET signal in the entorhinal cortex (TMTL^+^) compared with healthy younger (aged <39 years) controls. Compared with A^−^ TMTL^−^, A^−^ TMTL^+^ participants showed statistically significant, albeit moderate, longitudinal (mean [SD], 1.83 [0.84] years) tau PET increases that were largely limited to the temporal lobe, whereas those with A^+^ TMTL^+^ showed faster and more cortically widespread tau PET increases. In contrast to participants with A^+^ TMTL^+^, those with A^−^ TMTL^+^ did not show any noticeable Aβ accumulation over follow-up (mean [SD], 2.36 [0.76] years). Complementary cerebrospinal fluid analysis confirmed longitudinal p-tau181 increases in A^−^ TMTL^+^ in the absence of increased Aβ accumulation. Participants with A^−^ TMTL^+^ had accelerated MTL atrophy, whereas those with A^+^ TMTL^+^ showed accelerated atrophy in widespread temporoparietal brain regions. Increased MTL tau PET uptake in A^−^ individuals was associated with cognitive decline, but at a significantly slower rate compared with A^+^ TMTL^+^.

**Conclusions and Relevance:**

In this study, individuals with A^−^ TMTL^+^ exhibited progressive tau accumulation and neurodegeneration, but these processes were comparably slow, remained largely restricted to the MTL, were associated with only subtle changes in global cognitive performance, and were not accompanied by detectable accumulation of Aβ biomarkers. These data suggest that individuals with A^−^ TMTL^+^ are not on a pathologic trajectory toward AD.

## Key Points

**Question:**

What is the longitudinal trajectory of older individuals who show positron emission tomography–assessed medial temporal lobe (MTL) tau deposition in the absence of amyloid-β (Aβ) pathology (A^−^ TMTL^+^)?

**Findings:**

In this cohort study of 969 older participants, A^− ^TMTL^+^ individuals displayed moderate tau accumulation mainly restricted to the MTL, which was paralleled by cerebrospinal fluid phosphorylated tau increases and colocalized atrophy progression; no significant Aβ accumulation was observed. By contrast, Aβ-positive individuals showed pronounced and cortically widespread tau accumulation, which was accompanied by extratemporal cortical atrophy and significantly faster cognitive decline.

**Meaning:**

The findings of this study suggest that individuals with A^−^ TMTL^+^ do not appear to be on a pathologic trajectory toward Alzheimer disease.

## Teaser

This cohort study examines the pathologic and cognitive changes in older individuals with tau deposition in the medial temporal lobe noted on positron emission tomography without evidence of amyloid-β pathology.

## Introduction

Amyloid-β (Aβ) plaques and tau neurofibrillary tangles are the hallmarks of Alzheimer disease (AD).^1,2^ The presence of neurofibrillary tangles has been observed to be tightly linked to increased Aβ load.^3^ However, the presence of neurofibrillary tangles in the medial temporal lobe (MTL) has also been observed in older individuals without substantial Aβ pathology,^4^ a condition that has been termed *primary age-related tauopathy* (PART).^5^ Over recent years, clinicopathologic association studies have shed light on the clinical and neurodegenerative correlates of PART.^6,7,8,9,10^ Yet, being a neuropathologic entity that is only diagnosed at autopsy, little is known about the temporal course of this condition, and its association with downstream Aβ accumulation and the AD continuum^11^ remains controversial.^12,13,14^

Positron emission tomography (PET) studies have also consistently shown increased MTL tau PET signal in a subset of individuals with negative Aβ PET scans,^15,16,17^ which may reflect PART, among other possible conditions.^18^ The in vivo PET-based identification of these individuals also allows studying their future clinical and pathologic progression.

In this study, we used data from a large multicohort sample to study the longitudinal pathologic characteristics and future clinical course of Aβ PET-negative (A^−^) individuals who show increased MTL tau PET signal (TMTL^+^). Specifically, we studied baseline characteristics and longitudinal changes in cognition, neuroimaging, and cerebrospinal fluid (CSF) biomarkers in these individuals and contrasted them to biomarker-negative controls as well as to individuals with an AD-typical Aβ- and tau-positive PET profile.

## Methods

### Study Design

Data used in the preparation of this article were obtained from the Alzheimer’s Disease Neuroimaging Initiative (ADNI), Harvard Aging Brain Study (HABS),^19^ and AVID-A05 study cohorts (eMethods in Supplement 1). Informed written consent was obtained from all participants or their corresponding caregivers. All protocols were approved by each cohort’s respective institutional ethical review board. This study followed the Strengthening the Reporting of Observational Studies in Epidemiology (STROBE) reporting guideline. Data were collected between July 2, 2015, and August 23, 2021. We included all participants who had undergone concurrent structural magnetic resonance imaging, Aβ PET, tau PET, and clinical evaluation within a 6-month window (N = 1093). Participants were further classified into 4 groups according to PET-based Aβ (A) and tau (T) status, as described in the Neuroimaging section: A^−^ TMTL^−^ (n = 250), A^−^ TMTL^+^ (n = 264), A^+^ TMTL^+^ (n = 451), and A^+^ TMTL^−^ (n = 128). Additionally, a subcohort of 16 healthy younger controls (maximum age, <39 years) with concurrent magnetic resonance imaging and tau PET scans from the AVID-A05 study was included for the definition of the tau PET positivity threshold.

A subset from the ADNI study had baseline and follow-up CSF biomarkers available (described in the CSF Biomarkers section), and all participants had baseline cognitive data. Subsets of the study participants underwent follow-up neuroimaging (mean [SD], 2.36 [0.76] years for Aβ PET and 1.83 [0.84] years for tau PET) and cognitive assessments (eMethods in Supplement 1). Participants’ characteristics are provided in the Table.

**Table.** Cohort Characteristics

Characteristic	A^−^ TMTL^−^ (n = 250)	A^−^ TMTL^+^ (n = 264)	A^+^ TMTL^+^ (n = 451)
Study, No. (%)			
ADNI	128 (51.2)	178 (67.4)	330 (73.2)
HABS	65 (26.0)	52 (19.7)	36 (8.0)
AVID-A05	57 (22.8)	34 (12.9)	85 (18.8)
Age, mean (SD), y	70.0 (7.8)	74.9 (7.6)	75.6 (8.0)
Gender, No. (%)			
Men	108 (43.2)	133 (50.4)	221 (49.0)
Women	142 (56.8)	131 (49.6)	230 (51.0)
Years of education, mean (SD)	15.96 (2.80)	16.73 (2.60)	16.43 (2.47)
APOE-ε4 carrier, No. (%)^a^	49 (19.9)	45 (18.0)	236 (54.4)
APOE-ε2 carrier, No. (%)^a^	42 (16.3)	36 (14.4)	19 (4.4)
Cognitive status, No. (%)			
CU	189 (75.6)	175 (66.3)	180 (39.9)
MCI	55 (22.0)	71 (26.9)	172 (38.1)
ADD	6 (2.4)	18 (6.8)	99 (22.0)
MMSE score, mean (SD)	28.9 (1.59)	28.6 (1.93)	26.9 (3.60)
CU PACC-3, mean (SD)	0.25 (1.92)	−0.17 (2.30)	−0.27 (2.23)
CI ADAS-Cog 11, mean (SD)	9.85 (5.50)	10.3 (5.4)	13.9 (7.4)
Baseline biomarkers, mean (SD)			
Centiloids	−0.72 (7.76)	0.46 (7.89)	68.52 (37.31)
Braak stages I/II FTP SUVR	1.10 (0.09)	1.42 (0.35)	1.80 (0.56)
Braak stages III/IV FTP SUVR	1.19 (0.08)	1.30 (0.11)	1.72 (0.67)
Braak stages V/VI FTP SUVR	1.07 (0.08)	1.15 (0.10)	1.37 (0.44)
Log CSF Aβ42/40	−2.47 (0.19)	−2.50 (0.22)	−3.20 (0.41)
Log CSF p-tau181, pg/mL	2.80 (0.32)	2.91 (0.31)	3.32 (0.47)
Longitudinal biomarkers and cognition, yearly rates of change (SE)			
Centiloids	−0.17 (0.55)	0.0132 (0.6)	3.04 (1.98)
Braak stages I/II FTP SUVR	0.01 (0.02)	0.02 (0.02)	0.06 (0.05)
Braak stages III/IV FTP SUVR	0.01 (0.01)	0.02 (0.01)	0.07 (0.07)
Braak stages V/VI FTP SUVR	0.01 (0.01)	0.01 (0.01)	0.04 (0.05)
CU PACC-3^b^	−0.06 (0.20)	−0.09 (0.20)	−0.14 (0.25)
CI ADAS-Cog 11	1.30 (2.71)	1.61 (2.21)	3.11 (3.28)
Log CSF Aβ42/40 (1/y)	−0.0027 (0.0005)	−0.0027 (0.0007)	−0.0034 (0.0006)
Log CSF p-tau181, pg/mL/y	0.011 (0.013)	0.023 (0.022)	0.023 (0.017)

Abbreviations, ADAS-Cog 11, Alzheimer’s Disease Assessment Scale–Cognitive Subscale; ADD, Alzheimer disease dementia; ADNI, Alzheimer’s Disease Neuroimaging Initiative; APOE, apolipoprotein E; CI, cognitive impairment; CSF, cerebrospinal fluid; CU, cognitive unimpairment; FTP, [^18^F]flortaucipir; HABS, Harvard Aging Brain Study; MCI, mild cognitive impairment; MMSE, Mini-Mental State Examination; p-tau181, tau phosphorylated at threonine 181; PACC-3, Preclinical Alzheimer Cognitive Composite; SUVR, standardized uptake value ratio.

^a^ Only 915 participants had available APOE data (A^−^ TMTL^−^, 241; A^−^ TMTL^+^, 245; A^+^ TMTL^+^, 429).

^b^ Sum of the *z* scores of the MMSE total score, Log-Transformed Trail Test B, and Logical Memory Delayed Recall.

### Neuroimaging

Magnetic resonance imaging acquisition details for ADNI, HABS, and AVID-A05 are reported in the eMethods in Supplement 1. Magnetic resonance images were segmented with FreeSurfer, version 7.1.1 and Statistical Parametric Mapping 12 (SPM12, Wellcome Department of Imaging Neuroscience, Institute of Neurology). FreeSurfer-derived regions of interest (ROI) were merged to generate masks resembling regions affected by neurofibrillary tangle pathology in Braak stages I/II, III/IV, and V/VI (eMethods in Supplement 1).^20,21^ FreeSurfer-based cortical thickness maps were coregistered to the fsaverage template and smoothed with a 2-dimensional isotropic gaussian filter of 12 mm full width at half maximum.

PET acquisitions followed study-specific protocols that are detailed in the eMethods in Supplement 1. Tau-PET scans were acquired using [^18^F]flortaucipir (FTP), and Aβ-PET scans were acquired using either [^18^F]florbetapir (ADNI and AVID-A05), [^18^F]florbetaben (ADNI), or [^11^C]Pittsburgh compound B (HABS) radiotracers. The multicentric PET scans were preprocessed using an in-house-developed pipeline that replicated the ADNI pipeline for PET scanner harmonization.^22,23^ Scanner-specific gaussian filters were applied to each PET image (regardless of PET imaging modality) to reach a uniform isotropic resolution of 8 mm.

For FTP-PET scans, region-based voxelwise^24^ partial volume correction was applied using the PETPVC toolbox^25^ and Baker atlas.^26^ Global standardized uptake value ratio (SUVR) in Aβ-PET scans was quantified using the centiloid scale^27^ (eMethods in Supplement 1). In addition, cortical surface SUVR maps were generated for all PET scans using FreeSurfer,^28,29^ coregistered to the fsaverage template, and smoothed with a 2-dimensional isotropic gaussian filter of 10 mm full width at half maximum.

To minimize the effect of subthreshold Aβ burden in the A^−^ TMTL^+^ study group,^30,31,32^ Aβ positivity was defined using a conservative cutoff of 12 centiloids.^27^ This cut point proved to optimally discriminate between Thal phases 0 to 1 and 2 to 5^33^ and it is therefore lower compared with traditional cut points based on discrimination of AD neuropathologic change levels (24.4 centiloids^33^) or reliable worsening (19 centiloids^34^). The tau-positivity threshold was defined as the 95th percentile of regional entorhinal cortex (ERC) SUVR values in the younger control cohort^34^ (SUVR = 1.21) (eFigure 1 in Supplement 1).

### CSF Biomarkers

Cerebrospinal fluid samples were collected for a subset of ADNI participants and processed according to previously described protocols.^35^ Concentrations of Aβ1-42, Aβ1-40, and tau phosphorylated at threonine 181 (p-tau181) were measured by the ADNI Biomarker Core using the Roche Elecsys β-amyloid(1-42), β-amyloid(1-40), and phospho-tau (181P) CSF immunoassays. The CSF metrics used in this study included the baseline Aβ42/40 ratio (n = 359) and p-tau181 (n = 485) concentrations, as well as follow-up measurements for a subset of individuals (Aβ42/40: n = 77; mean [SD], 2.30 [1.03] years; p-tau181: n = 99; 2.34 [1.05] years).

### Cognitive Assessments

Cognitive performance in cognitively unimpaired individuals was assessed using a modified version of the Preclinical Alzheimer Cognitive Composite^36^ (PACC) derived as the sum of the *z* scores of the Mini-Mental State Examination total score, Log-Transformed Trail Test B, and Logical Memory Delayed Recall (PACC-3). The PACC-3 is designed to detect the first signs of cognitive decline in otherwise asymptomatic individuals. Cognitive performance in cognitively impaired individuals (combined mild cognitive impairment and AD dementia) was assessed using the Alzheimer’s Disease Assessment Scale–Cognitive Subscale (ADAS-Cog 11).

### Statistical Analysis

Statistical analysis of differences between the A^−^ TMTL^−^ vs A^−^ TMTL^+^ and A^+^ TMTL^+^ study groups was performed using generalized linear models (GLMs) controlled for age, sex, cohort (ADNI, HABS, and AVID-A05), and baseline centiloid values in the case of A^−^ TMTL^+^ vs A^−^ TMTL^−^ comparisons. Effect sizes were measured using Cohen *d*, and group differences between cortical maps were corrected for multiple comparisons using the FreeSurfer clusterwise correction for multiple comparisons. Longitudinal rates of change were computed using linear mixed-effect models with participant-specific intercepts and slopes (eg, V_k_ ~ time + (time|participant), where V_k_ is the value on the kth vertex of a cortical map).

First, we investigated vertex-wise and ROI-based group differences in baseline FTP SUVRs. Vertex and ROI-based group differences were also computed for the FTP SUVR longitudinal rates of change. Additionally, group differences in longitudinal centiloid accumulation were similarly investigated. Analysis of baseline and longitudinal differences in CSF Aβ42/40 and p-tau181 biomarker levels used analogous statistical models, but values were log-transformed before analysis to account for the exponential progression of CSF biomarker levels. Baseline and longitudinal differences across groups in cognitive metrics were studied separately for cognitively unimpaired and cognitively impaired individuals because of the different neuropsychological instruments that are best suited to detect the subtle cognitive changes in participants without impairment and more overt cognitive changes in those with impairment. As post hoc sensitivity analyses, we repeated the previous analyses with higher cut points for Aβ (24 centiloids) and tau PET positivity (mean +2.5 SD of the ERC FTP, SUVR = 1.27). Moreover, we assessed the outcome of using a larger MTL ROI comprising the ERC and amygdala.

In addition to the comparisons of dichotomized A and TMTL groups, complementary analyses were performed to assess continuous associations of baseline ERC FTP SUVR with vertex-wise cortical thickness patterns across all A^−^ individuals, using GLMs adjusted by sex, age, cohort, and baseline centiloid. Analogously, associations between baseline ERC FTP SUVR and cognitive performance were studied across the A^−^ subcohort with equally adjusted GLMs. Statistical tests were 2-sided, and *P* < .05 was considered statistically significant. The strength of the associations was assessed using the Pearson partial correlation coefficient (*r*).

## Results

### Demographic Characteristics

Of the 965 individuals included in the study, 462 were men (47.9%) and 503 were women (52.1%); mean (SD) age was 73.9 (8.1) years. A total of 51% A^−^ individuals and 78% of A^+^ participants had increased tau PET signal in the ERC (TMTL^+^) compared with healthy younger (age, <39 years) controls. Further demographic and biomarker characteristics are reported in the Table. Of participants with race data available (ie, ADNI and HABS cohorts), 92.9% of the individuals were White. Although no significant differences between women and men were found in baseline Braak stages I/II FTP-PET SUVR (*d* = 0.10; 95% CI, −0.02 to 0.23; *P* = .10) (eFigure 2 in Supplement 1), slightly higher longitudinal rates of Braak stages I/II FTP-PET SUVR change were observed in women (*d* = 0.13; 95% CI, 0.02-0.23; *P* = .02). Both A^−^ TMTL^+^ (mean [SD] age, 74.9 [7.6] years; *d* = 0.64; 95% CI, 0.47-0.83; *P* < .001) and A^+^ TMTL^+^ (age, 75.6 [8.0] years; *d* = 0.70; 95% CI, 0.55-0.86, *P* < .001) individuals were significantly older than the A^−^ TMTL^−^ control cohort (age, 70.0 [7.8] years). Similarly, both the A^+^ TMTL^+^ (60.4%; *d* = 0.69; 95% CI, 0.56-0.80; *P* < .001) and A^−^ TMTL^+^ (33.7%; *d* = 0.23; 95% CI, 0.06-0.40; *P* = .009) groups had a significantly higher proportion of cognitively impaired individuals than the A^−^ TMTL^−^ group (24.4%). The prevalence of apolipoprotein E (APOE)–ε4 was higher among A^+^ TMTL^+^ individuals (54.4%, *d* = 0.64; 95% CI, 0.54-0.76; *P* < .001), but was similar between the A^−^ TMTL^+^ (18.0%; *d* = −0.01; 95% CI, −0.19 to 0.16; *P* = .68) and the A^−^ TMTL^−^ control group (19.9%). By contrast, both the A^−^ TMTL^−^ (16.3%) and A^−^ TMTL^+^ groups (14.4%; *d* = 0.08; 95% CI, −0.10 to 0.25; *P* = .38) showed significantly higher proportions of APOE-ε2 carriers than the A^+^ TMTL^+^ group (4.4%; *d* = 0.38; 95% CI, 0.24-0.49; *P* < .001).

### Tau and Aβ Accumulation

**Figure 1.**
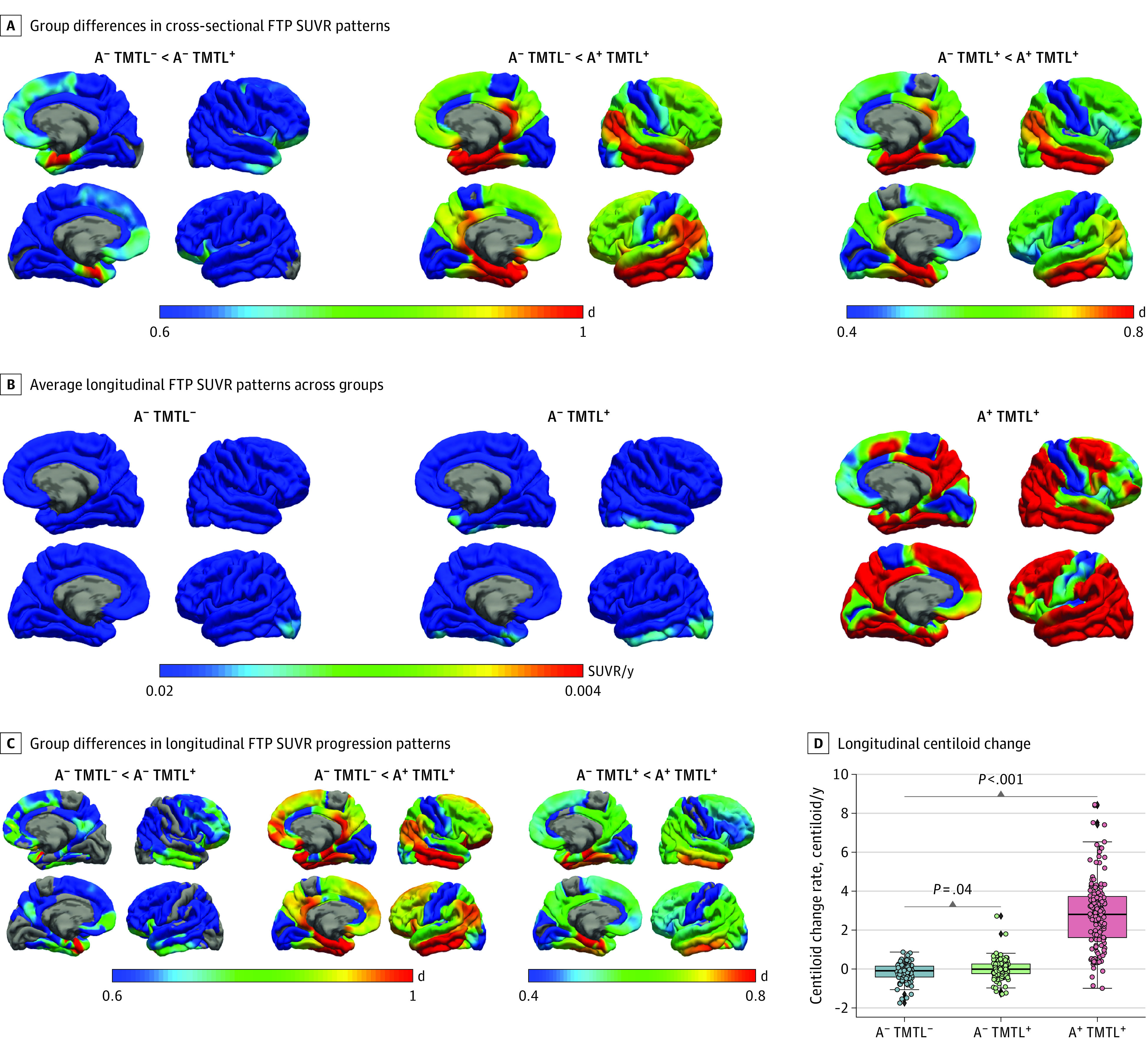
Cross-Sectional and Longitudinal Characterization of Amyloid-β (A) and Tau (T) Positron Emission Tomography Accumulation in the Medial Temporal Lobe (MTL) A, Vertex-wise group differences in cross-sectional [^18^F]flortaucipir (FTP) standardized uptake value ratio (SUVR) patterns in A^−^ TMTL^+^ and A^+^ TMTL^+^ individuals compared with the control group, expressed as Cohen *d*. B, Average longitudinal FTP SUVR patterns in A^−^ TMTL^−^, A^−^ TMTL^+^, and A^+^ TMTL^+^ individuals, represented as vertex-wise rates of changes. C, Vertex-wise group differences in longitudinal FTP SUVR in A^−^ TMTL^+^ and A^+^ TMTL^+^ individuals compared with the A^−^ TMTL^−^ control group, expressed as Cohen *d*. D, Longitudinal centiloid change in A^−^ TMTL^−^, A^−^ TMTL^+^, and A^+^ TMTL^+^ individuals.

Analysis of baseline FTP SUVR contrast maps (Figure 1A) noted increased tau burden in A^−^ TMTL^+^ individuals to be most pronounced in the MTL and extending into the inferior temporal lobe and the ventromedial prefrontal cortex, while A^+^ TMTL^+^ individuals showed the AD-characteristic pattern of widespread cortical tau accumulation across temporal, parietal, and frontal areas. In vertex-wise longitudinal FTP SUVR analyses, A^−^ TMTL^−^ individuals showed little increase of tau accumulation over time, whereas the A^−^ TMTL^+^ cohort displayed a moderate increase of tau uptake restricted to the MTL and inferior temporal regions (Figure 1B). By contrast, A^+^ TMTL^+^ participants showed a pronounced and widespread increase of tau accumulation. These differences were confirmed in direct statistical contrasts between the TMTL^+^ groups and the A^−^ TMTL^−^ group (Figure 1C). An ROI-based FTP SUVR analysis showcased similar results (eFigure 3 in Supplement 1), with A^−^ TMTL^+^ participants showing statistically significant albeit moderate longitudinal (mean [SD], 1.83 [0.84] years) tau PET increases that were largely limited to the temporal lobe, whereas those with A^+^ TMTL^+^ showed faster and more cortically widespread tau PET increases.

Regarding Aβ accumulation, centiloid rates of change in A^−^ TMTL^+^ participants did not show any significant increase in centiloids over time (mean [SD], 0.01 [12]; *P* = .82) (eFigure 4 in Supplement 1), although the slopes were slightly different from the slopes of the A^−^ TMTL^−^ group (−0.17 [0.55]; *d* = 0.29; 95% CI, 0.04 to 0.54; *P* = .04) (Figure 1D). By contrast, A^+^ TMTL^+^ individuals showed a pronounced increase in centiloids over time (3.04 [1.98] vs −0.17 [0.55]; *d* = 1.89; 95% CI, 1.64 to 2.24; *P* < .001).

**Figure 2.**
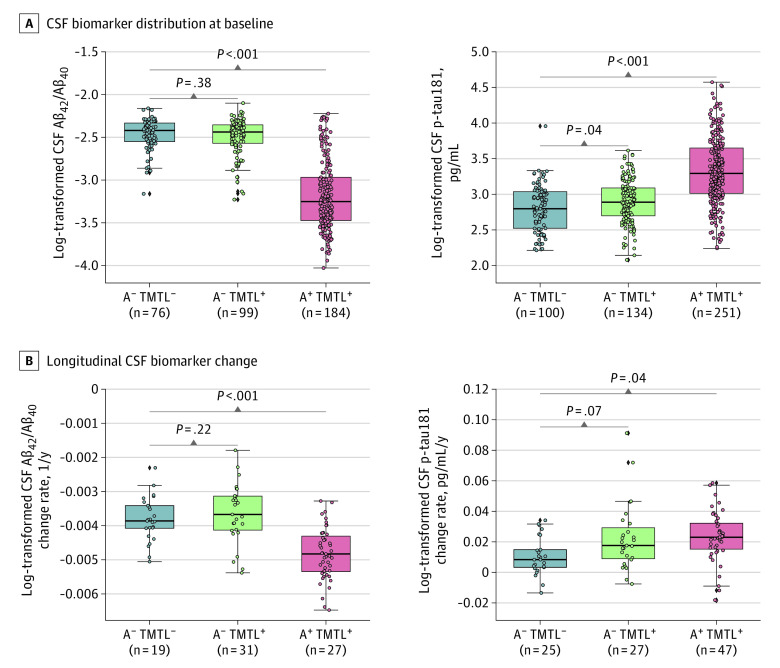
Baseline and Longitudinal Characterization of Cerebrospinal Fluid (CSF) Amyloid-β (Aβ) and Tau Biomarkers A, Baseline levels of CSF Aβ42/40 and tau phosphorylated at threonine 181 (p-tau181) across groups. Biomarker levels were statistically compared using generalized linear models (GLMs). B, Longitudinal change of CSF Aβ42/40 and p-tau181 metrics across groups, obtained using linear mixed-effect models and statistically compared with GLMs.

In the CSF subset analysis, A^−^ TMTL^+^ participants showed moderately higher baseline p-tau181 levels compared with the A^−^ TMTL^−^ group (*d* = 0.24; 95% CI, 0.002-0.48; *P* = .04), but no significant difference in Aβ42/40 (*d* =  −0.10; 95% CI, −0.34 to 0.14; *P* = .38) (Figure 2A), whereas A^+^ TMTL^+^ individuals exhibited the expected alterations in both Aβ42/40 (*d* =  −1.38; 95% CI, −1.66 to −1.14; *P* < .001) and p-tau181 (*d* = 1.00; 95% CI, 0.81-1.20; *P* < .001) levels (Figure 2A). In longitudinal analyses, the A^−^ TMTL^+^ group showed larger increases in p-tau181 levels over time at trend-level statistical significance (*d* = 0.52; 95% CI, 0.08-1.04; *P* = .07), but no significant difference in Aβ42/40 ratio change (*d* = 0.34; 95% CI, −0.16 to 0.94; *P* = .22), compared with the A^−^ TMTL^−^ group (Figure 2B). The A^+^ TMTL^+^ group showed significantly faster rates of change in both biomarkers (Aβ42/40: *d* = −1.33; 95% CI, −1.92 to −0.87; *P* < .001; p-tau181: *d* = 0.53; 95% CI, 0.07-1.06; *P* = .04).

### Neurodegeneration

**Figure 3.**
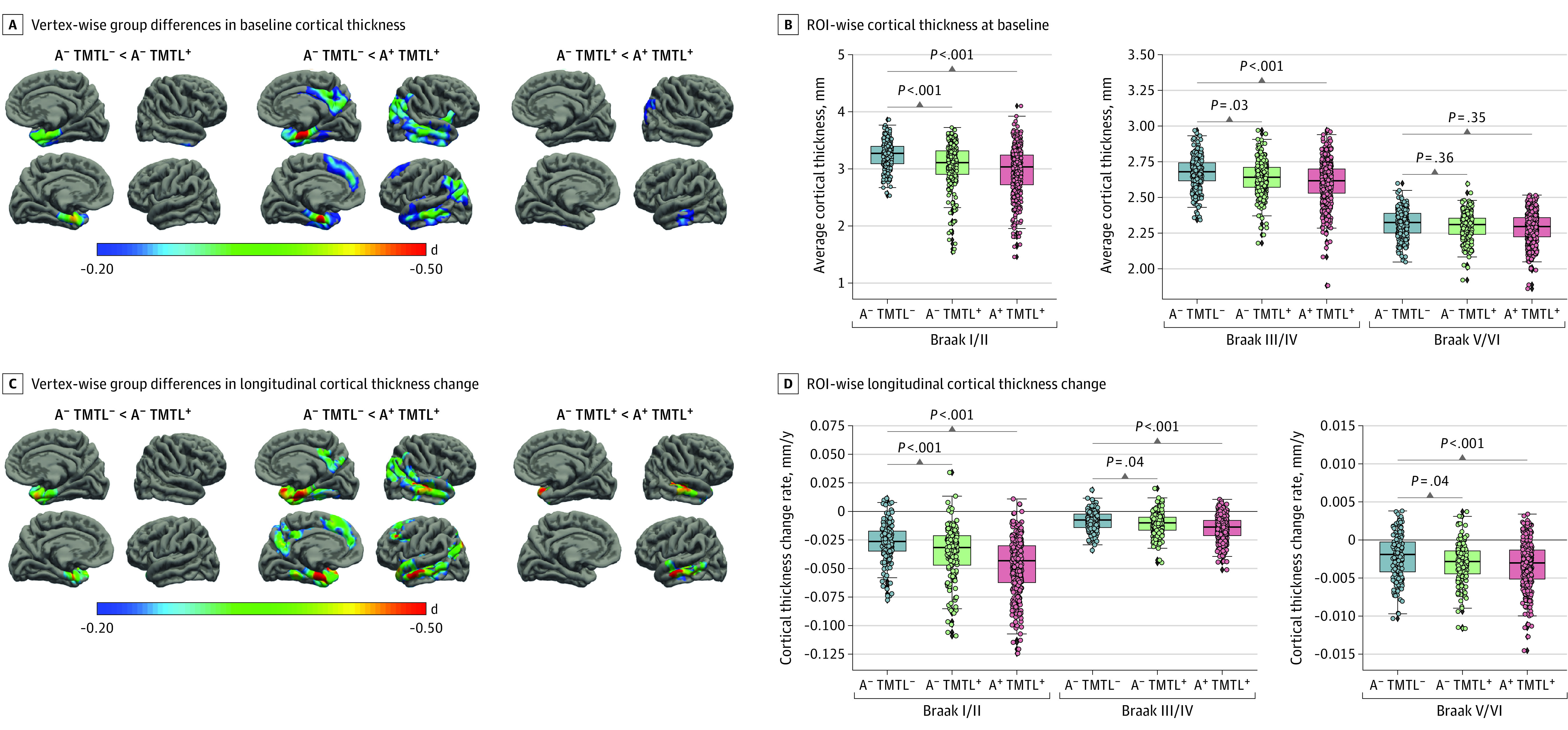
Baseline and Longitudinal Characterization of Atrophy, as Shown With Structural Magnetic Resonance Imaging A, Vertex-wise group differences in baseline cortical thickness patterns in A^−^ TMTL^+^ and A^+^ TMTL^+^ individuals compared with the A^−^ TMTL^−^ control group, measured as Cohen *d*. B, Regional (region of interest [ROI]) group differences in baseline cortical thickness patterns in A^−^ TMTL^+^ (Braak stages I/II: *d* = −0.50; 95% CI, −0.65 to −0.34; *P* < .001; Braak stages III/IV: *d* = −0.20; 95% CI, −0.38 to −0.02; *P* = .025; Braak stages V/VI: *d* = −0.08; 95% CI, −0.26 to 0.10; *P* = .36), and A^+^ TMTL^+^ individuals (Braak stages I/II: *d* = −0.60; 95% CI, −0.73 to −0.46; *P* < .001; Braak stages III/IV: *d* = −0.34; 95% CI, −0.49 to −0.20; *P* < .001; Braak stages V/VI: *d* = −0.17; 95% CI, −0.32 to −0.02; *P* = .035) compared with the A^−^ TMTL^−^ control group using generalized linear models (GLM). C, Vertex-wise group differences in longitudinal cortical thickness progression patterns in A^−^ TMTL^+^, and A^+^ TMTL^+^ individuals compared with the A^−^ TMTL^−^ control group, measured as Cohen *d*. D, Regional group differences in longitudinal cortical thickness progression patterns in A^−^ TMTL^+^ (Braak stages I/II: *d* = −0.38; 95% CI, −0.60 to −0.16; *P* < .001; Braak stages III/IV: *d* = −0.22; 95% CI, −0.44 to 0.008; *P* = .044; Braak stages V/VI: *d* = −0.22; 95% CI, −0.44 to −0.005; *P* = .041), and A^+^ TMTL^+^ individuals (Braak stages I/II: *d* = −0.76; 95% CI, −0.95 to −0.59; *P* < .001; Braak stages III/IV: *d* = −0.57; 95% CI, −0.76 to −0.39; *P* < .001; Braak stages V/VI: *d* = −0.34; 95% CI, −0.53 to −0.16; *P* < .001) compared with the A^−^ TMTL^−^ control group using GLM models.

Compared with the A^−^ TMTL^−^ group, A^−^ TMTL^+^ participants (Figure 3A) showed cortical thinning at baseline mainly restricted to the MTL, whereas A^+^ TMTL^+^ participants showed more widespread cortical thinning extending to the lateral temporal lobe, the posterior cingulate, and the parietal and frontal lobes. This pattern was also reflected in ROI-based analyses, with A^−^ TMTL^+^ individuals showing significant cortical thinning in Braak stages I/II and Braak stages III/IV only (Figure 3B). The complementary analysis using continuous tau PET measures confirmed an association between ERC FTP SUVR and medial temporal neurodegeneration across A^−^ individuals (eFigure 5 in Supplement 1).

In longitudinal analyses, A^−^ TMTL^+^ individuals showed faster cortical thinning compared with the A^−^ TMTL^−^ group that was largely restricted to the MTL, while accelerated cortical thinning in the A^+^ TMTL^+^ group further extended to the lateral temporal, parietal, and frontal lobes (Figure 3C). Similarly, ROI-wise analyses (Figure 3D) showed significantly faster cortical thinning in A^−^ TMTL^+^ participants, mostly in Braak stages I/II.

### Cognition

**Figure 4.**
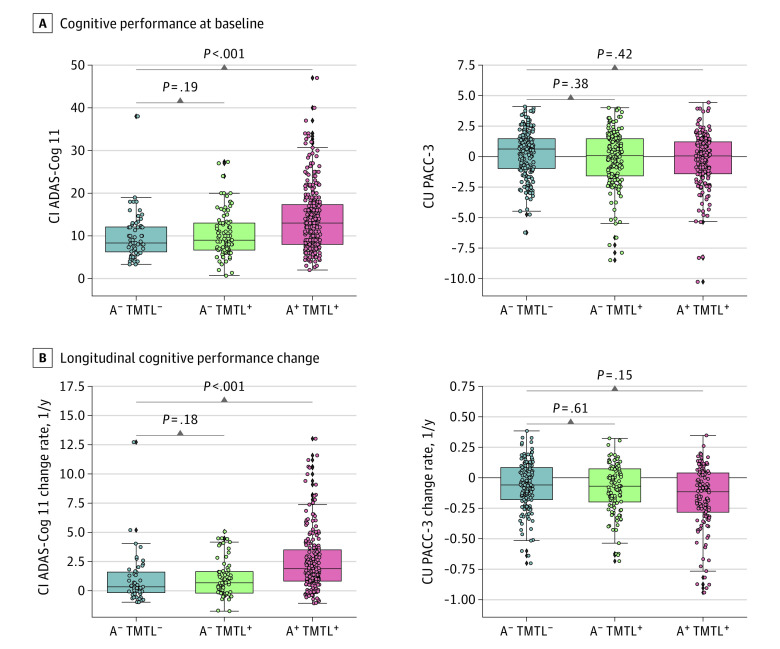
Baseline and Longitudinal Characterization of Cognitive Performance A, Baseline Alzheimer’s Disease Assessment Scale–Cognitive Subscale (ADAS-Cog 11) scores in cognitively impaired (CI) individuals and sum of the *z* scores of the Mini-Mental State Examination total score, Log-Transformed Trail Test B, and Logical Memory Delayed Recall scores (PACC-3) in cognitively unimpaired (CU) individuals across amyloid-β (A) and tau (T) medial temporal lobe (MTL) groups, compared using generalized linear models (GLMs). B, Longitudinal changes in ADAS-Cog 11 scores in CI individuals and PACC-3 scores in CU individuals across A and TMTL groups, obtained with linear mixed-effect models and compared using GLMs.

At baseline, cognitively impaired A^+^ TMTL^+^ individuals showed lower ADAS-Cog 11 scores compared with the cognitively impaired A^−^ TMTL^−^ group (*d* = −0.57; 95% CI, −0.79 to −0.34; *P* < .001), but neither the cognitively impaired A^−^ TMTL^+^ individuals nor any of the cognitively unimpaired groups differed significantly from the respective A^−^ TMTL^−^ controls (Figure 4). In the complementary analysis with continuous tau PET measures, baseline ERC FTP SUVR was significantly correlated with worse cognition in cognitively impaired A^−^ individuals (ADAS-Cog 11: *r* = 0.26; 95% CI, 0.08-0.46; *P* = .001), but not in cognitively unimpaired A^−^ individuals (PACC-3: *r* = 0.02; 95% CI, −0.09 to 0.13; *P* = .72).

Longitudinally, cognitively impaired individuals with A^−^ TMTL+ showed a comparable degree of moderate decline in ADAS-Cog 11 scores compared with cognitively impaired A^−^ TMTL^−^ individuals (*d* = 0.25; 95% CI, −0.11 to 0.58; *P* = .18), whereas cognitively impaired A^+^ TMTL^+^ participants showed significantly faster cognitive deterioration (*d* = 0.60; 95% CI, 0.30-0.90; *P* < .001) (eFigure 6 in Supplement 1). Among cognitively unimpaired participants, neither A^−^ TMTL^+^ (*d* = −0.06; 95% CI, −0.25 to 0.40; *P* = .61) nor A^+^ TMTL^+^ (*d* = −0.17; 95% CI, −0.44 to 0.14; *P* = .15) individuals showed a significant difference in PACC-3 decline compared with the A^−^ TMTL^−^ group. In the complementary analysis with continuous tau PET measures, baseline ERC FTP SUVR was significantly correlated with faster cognitive decline in cognitively impaired A^−^ individuals (ADAS-Cog 11: *r* = 0.30; 95% CI, 0.15-0.53; *P* < .001), but not in cognitively unimpaired A^−^ individuals (PACC-3: *r* = 0.03; 95% CI, −0.22 to 0.14; *P* = .63).

### Sensitivity Analyses

Overall, the results derived from the sensitivity analyses were consistent with the main results presented in this study. Similar patterns of tau PET SUVR, CSF biomarkers, atrophy, and clinical change were found across the A TMTL groups when changing the Aβ PET cut point to 24 centiloids (eFigures 7-10 in Supplement 1) and when changing the Braak stages I/II SUVR cut point to 1.27 (eFigures 11-14 in the Supplement 1). Analyses using a larger MTL ROI (ERC plus amygdala) yielded a slightly different distribution of A TMTL groups (eFigure 15 in Supplement 1) and showed that the Aβ- and tau- accumulation patterns were similar to those obtained with the ERC ROI.

## Discussion

In this study, we explored in detail the pathologic and clinical course of older individuals who display PET-measured tau accumulation in the MTL in the absence of Aβ pathology (A^−^ TMTL^+^), a condition reminiscent of pathologically defined PART.^5^ In a large multicentric cohort of almost 1000 older individuals, we found that increased MTL tau PET signal without notable Aβ pathology is relatively common in older individuals and is associated with further longitudinal tau PET uptake increase, which remains largely restricted to the MTL. These tau PET increases colocalize with progressive MTL neurodegeneration, are associated with only subtle changes in global cognitive performance, and are not accompanied by notable accumulation of Aβ pathology over time.

Using a tau PET cutoff defined in healthy younger individuals, we observed that tau PET-measured MTL accumulation in the absence of Aβ is a common condition in older individuals, representing 51% of A^−^ individuals in this study. The frequency of tau PET positivity in this A^−^ sample is consistent with a previous study using a similar method (67%),^17^ and it is substantially higher compared with previous studies using larger temporal ROIs without partial volume correction (approximately 17%-20%).^15,37^ The discrepancy may be explained by use of extra-MTL ROIs without partial volume correction, which results in a lack of sensitivity to MTL-specific signal.

The degree to which FTP-PET can detect PART remains a subject of debate. PET-to-autopsy studies generally agree that local tau pathology needs to reach a certain density of neurofibrillary tangles to be detected in an FTP-PET scan, which is mostly the case for Braak stages V/VI.^38,39,40,41^ This may lead to the conclusion that FTP-PET cannot detect PART-related tau deposition, which is, by definition, Braak stage IV or less. Yet, FTP showed binding to neurofibrillary tangles from PART brains in autoradiography studies^42,43^ and, therefore, FTP-PET may detect a subset of PART cases with suprathreshold neurofibrillary tangle density. To date, the number of PART cases in the available PET-to-autopsy studies is low (n = 3)^38^ and we cannot exclude that PART could be detected with FTP-PET in a subset of individuals. This hypothesis is consistent with the fact that the prevalence of tau PET positivity among older A^−^ individuals in our study (51%) is considerably lower than the prevalence of PART in this age range in neuropathologic studies.^5^ The topography of our findings is also consistent with PART: in line with recent studies,^15,44,45^ our results showed that increased tau PET signal in A^−^ TMTL^+^ individuals was largely limited to the MTL. Both baseline and longitudinal increases in tau PET signal in A^−^ TMTL^+^ individuals were found to be paralleled by increases in CSF p-tau181 levels, suggesting that these signal increases reflect actual increases in tau burden. Together, these results suggest that PART may be an important neuropathologic substrate for many A^−^ TMTL^+^ individuals in our study, although probably not the only one.^15^

We also acknowledge that pathologic entities other than PART may lead to abnormal FTP-PET signal in the MTL among Aβ-negative individuals. Although FTP shows high specificity for AD-type tau aggregates in autoradiography studies,^42,46,47^ extensive increases in cortical FTP-PET signal in the absence of Aβ can occur in patients with AD dementia or mild cognitive impairment, which likely represent tangle-predominant dementia.^15,48^ Moreover, FTP-PET increases can occur in frontotemporal dementia syndromes, including those associated with tau and TAR DNA-binding protein 43 (TDP-43).^49,50,51,52^ The binding mechanisms remain unclear, although binding to non-AD tau as well as to neurodegenerative processes that colocalize with TDP-43 deposition might result in nonspecific FTP binding.^49,53^ Therefore, we cannot exclude the possibility that limbic-predominant age-related TDP-43 encephalopathy, which is associated with neurodegeneration in the MTL, might also result in abnormal FTP-PET signal in the same regions, although the influence of limbic-predominant age-related TDP-43 encephalopathy on FTP-PET appears to be limited.^54^ These considerations suggest that the A^−^ TMTL^+^ group likely represents both a clinically and pathologically heterogeneous group. Thus, increased FTP-PET signal in the MTL in the absence of Aβ should not be considered a specific marker of PART. Despite these limitations, our findings are valuable and contribute to understanding the clinical and pathologic course of the A^−^ TMTL^+^ group as a whole. Yet, given its high frequency among cognitively unimpaired individuals and those with mild average cognitive decline, the clinical significance of the A^−^ TMTL^+^ profile remains uncertain. Additional work is needed to identify the subset of A^−^ TMTL^+^ individuals who will experience more relevant clinical outcomes.

Given that longitudinal follow-up of neuropathologically defined PART is not possible, a main controversy exists whether PART reflects an early form of AD, with Aβ pathology developing in the further disease course, or whether it represents an entirely distinct pathologic entity.^12,13,14^ Herein, we noted that A^−^ TMTL^+^ individuals did not show significant Aβ accumulation over the available follow-up period, thus arguing against the possibility that this condition reflects an early tau-first subtype of AD.^55^

Longitudinal cortical thickness analysis demonstrated that A^−^ TMTL^+^ individuals have moderate and restricted MTL atrophy progression, whereas atrophy is more accelerated and spreads to widespread neocortical regions in A^+^ TMTL^+^ participants. These results suggest that tau accumulation in Aβ-negative individuals is not a benign process but is associated with increased neurodegeneration,^56^ although the rates of progression are significantly slower compared with rates in A^+^ TMTL^+^ participants.

In cognition analyses, we did not find significant differences in baseline performance or longitudinal decline between A^−^ TMTL^+^ individuals and the A^−^ TMTL^−^ controls, whereas a significantly faster decline was observed in cognitively impaired A^+^ TMTL^+^ individuals. However, a more sensitive analysis using continuous tau measures showed an association of ERC tau uptake with worse cognition and faster cognitive decline also in cognitively impaired A^−^ participants. These results suggest that, in the absence of Aβ, tau accumulation in the MTL has only subtle effects on cognition and does not herald the pronounced cognitive decline typical for AD. Further research with longer follow-up might be necessary to delineate the long-term consequences of ongoing tau accumulation in the absence of Aβ.

### Limitations

This study has limitations. The first of these is the lack of autopsy data of A^−^ TMTL^+^ individuals, which leaves the exact association between the PET-defined A^−^ TMTL^+^ group and PART to be determined. Second, we relied on cutoffs for group definition. While centiloid cutoffs for denoting Aβ status are well established,^27^ a number of different methods and cutoffs for defining tau PET positivity have been used in the literature, resulting in highly variable proportions of the different A and TMTL groups.^57^ Herein, we applied a commonly used method for objectively defining biomarker cutoffs based on data from healthy younger controls,^34^ and several of our principal findings were replicated in complementary continuous analyses that are independent of cutoff definition. Third, to achieve robust sample sizes of the less-prevalent A^−^ TMTL^+^ individuals, we pooled data across different cohorts. While the possible influence of multicentric data acquisitions was minimized by harmonizing imaging preprocessing, it limited our ability to analyze domain-specific cognitive decline, as neuropsychological instruments differed across cohorts. Fourth, follow-up time for the evaluation of both longitudinal clinical and biomarker measures was relatively short. Fifth, the cohorts included in our study represent selective research cohorts that may not reflect the general population, and our findings should be replicated in more diverse cohorts.

## Conclusions

The results presented in this longitudinal cohort study suggest that individuals with MTL tau accumulation in the absence of Aβ follow a separate, less malign, pathologic course compared with that of typical AD. While these individuals showed progressive tau accumulation and neurodegeneration, this process was comparably slow, remained largely restricted to the MTL, and was associated with only subtle changes in global cognitive performance. Moreover, these individuals did not show notable Aβ accumulation over follow-up, arguing against the possibility that this A^−^ TMTL^+^ condition reflects a tau-first subtype of AD. Further studies are warranted that specify the exact association of this common PET-defined condition with pathologic PART.

## Notes

Supplement 1. **eMethods.** Participants and Study Design, Image Acquisition, Image Processing and Follow-up Data **eFigure 1.** MTL Tau PET SUVR Distribution Across Groups **eFigure 2.** MTL Tau-PET Positivity Across Age and Sex **eFigure 3.** Baseline Levels and Longitudinal Change Rates of ROI-Wise FTP-PET SUVR Values **eFigure 4.** Longitudinal Change From Baseline in Centiloids Across Groups **eFigure 5.** Association of Baseline ERC FTP SUVR With Baseline Cortical Thickness in A- Individuals **eFigure 6.** Longitudinal Change From Baseline in Group-Wise ADAS-Cog 11 Scores in CI Individuals **eFigure 7.** Cross-sectional and Longitudinal Tau- and Aβ-PET Accumulation, Using an Aβ-PET Positivity Cut-Point of 24 Centiloids **eFigure 8.** Baseline and Longitudinal CSF Aβ and Tau Biomarker Levels, Using an Aβ-PET Positivity Cut-Point of 24 Centiloids **eFigure 9.** Baseline and Longitudinal Cortical Thickness, Using an Aβ-PET Positivity Cut-Point of 24 Centiloids **eFigure 10.** Baseline and Longitudinal Cognitive Performance, Using an Aβ-PET Positivity Cut-Point of 24 Centiloids **eFigure 11.** Cross-sectional and Longitudinal Tau- and Aβ-PET Accumulation, Using a Tau-PET Positivity SUVR Cut-Point of 1.27 **eFigure 12.** Baseline and Longitudinal CSF Aβ and Tau Biomarker Levels, Using a Tau-PET Positivity SUVR Cut-Point of 1.27 **eFigure 13.** Baseline and Longitudinal Cortical Thickness, Using a Tau-PET Positivity SUVR Cut-Point of 1.27 **eFigure 14.** Baseline and Longitudinal Cognitive Performance, Using a Tau-PET Positivity SUVR Cut-Point of 1.27 **eFigure 15.** Cross-sectional and Longitudinal Tau- and Aβ-PET Accumulation, Using an Entorhinal Cortex & Amygdala ROI
eReferences

Supplement 2. **Nonauthor Collaborators.** The Alzheimer’s Disease Neuroimaging Initiative and the Harvard Aging Brain Study

Supplement 3.
Data Sharing Statement
