# Supplementary material for: Increased Medial Temporal Tau Positron Emission Tomography Uptake in the Absence of Amyloid-β Positivity
Source: JAMA Neurol. 2023 Aug 14;80(10):1051–61. doi: 10.1001/jamaneurol.2023.2560 (PMC10425864; doi:10.1001/jamaneurol.2023.2560)
Supplement: Supplement 1. — eMethods. Participants and Study Design, Image Acquisition, Image Processing and Follow-up Data eFigure 1. MTL Tau PET SUVR Distribution Across Groups eFigure 2. MTL Tau-PET Positivity Across Age and Sex eFigure 3. Baseline Levels and Longitudinal Change Rates of ROI-Wise FTP-PET SUVR Values eFigure 4. Longitudinal Change From Baseline in Centiloids Across Groups eFigure 5. Association of Baseline ERC FTP SUVR With Baseline Cortical Thickness in A- Individuals eFigure 6. Longitudinal Change From Baseline in Group-Wise ADAS-Cog 11 Scores in CI Individuals eFigure 7. Cross-sectional and Longitudinal Tau- and Aβ-PET Accumulation, Using an Aβ-PET Positivity Cut-Point of 24 Centiloids eFigure 8. Baseline and Longitudinal CSF Aβ and Tau Biomarker Levels, Using an Aβ-PET Positivity Cut-Point of 24 Centiloids eFigure 9. Baseline and Longitudinal Cortical Thickness, Using an Aβ-PET Positivity Cut-Point of 24 Centiloids eFigure 10. Baseline and Longitudinal Cognitive Performance, Using an Aβ-PET Positivity Cut-Point of 24 Centiloids eFigure 11. Cross-sectional and Longitudinal Tau- and Aβ-PET Accumulation, Using a Tau-PET Positivity SUVR Cut-Point of 1.27 eFigure 12. Baseline and Longitudinal CSF Aβ and Tau Biomarker Levels, Using a Tau-PET Positivity SUVR Cut-Point of 1.27 eFigure 13. Baseline and Longitudinal Cortical Thickness, Using a Tau-PET Positivity SUVR Cut-Point of 1.27 eFigure 14. Baseline and Longitudinal Cognitive Performance, Using a Tau-PET Positivity SUVR Cut-Point of 1.27 eFigure 15. Cross-sectional and Longitudinal Tau- and Aβ-PET Accumulation, Using an Entorhinal Cortex & Amygdala ROI eReferences [file jamaneurol-e232560-s001.pdf]

Supplementary Online Content

Costoya-Sánchez A, Moscoso A, Silva-Rodríguez J, et al; Alzheimer's Disease Neuroimaging Initiative and the Harvard Aging Brain Study. Increased medial temporal tau positron emission tomography uptake in the absence of amyloid-β positivity. *JAMA Neurol*. Published online August 14, 2023. doi:10.1001/jamaneurol.2023.2560

eMethods. Participants and Study Design, Image Acquisition, Image Processing and Follow-up Data

eFigure 1. MTL Tau PET SUVR Distribution Across Groups

eFigure 2. MTL Tau-PET Positivity Across Age and Sex

eFigure 3. Baseline Levels and Longitudinal Change Rates of ROI-Wise FTP-PET SUVR Values

eFigure 4. Longitudinal Change From Baseline in Centiloids Across Groups

eFigure 5. Association of Baseline ERC FTP SUVR With Baseline Cortical Thickness in A- Individuals

eFigure 6. Longitudinal Change From Baseline in Group-Wise ADAS-Cog 11 Scores in CI Individuals

eFigure 7. Cross-sectional and Longitudinal Tau- and Aβ-PET Accumulation, Using an Aβ-PET Positivity Cut-Point of 24 Centiloids

eFigure 8. Baseline and Longitudinal CSF Aβ and Tau Biomarker Levels, Using an Aβ-PET Positivity Cut-Point of 24 Centiloids

eFigure 9. Baseline and Longitudinal Cortical Thickness, Using an Aβ-PET Positivity Cut-Point of 24 Centiloids

eFigure 10. Baseline and Longitudinal Cognitive Performance, Using an Aβ-PET Positivity Cut-Point of 24 Centiloids

eFigure 11. Cross-sectional and Longitudinal Tau- and Aβ-PET Accumulation, Using a Tau-PET Positivity SUVR Cut-Point of 1.27

eFigure 12. Baseline and Longitudinal CSF Aβ and Tau Biomarker Levels, Using a Tau-PET Positivity SUVR Cut-Point of 1.27

eFigure 13. Baseline and Longitudinal Cortical Thickness, Using a Tau-PET Positivity SUVR Cut-Point of 1.27

eFigure 14. Baseline and Longitudinal Cognitive Performance, Using a Tau-PET Positivity SUVR Cut-Point of 1.27

eFigure 15. Cross-sectional and Longitudinal Tau- and Aβ-PET Accumulation, Using an Entorhinal Cortex & Amygdala ROI

eReferences

This supplementary material has been provided by the authors to give readers additional information about their work.

© 2023 Costoya-Sánchez A et al. *JAMA Neurology*.

eMethods. Participants and Study Design, Image acquisition, Image processing and Follow-Up Data

Participants and Study Design

ADNI

ADNI is an ongoing, longitudinal, multicenter study conducted in 59 sites across the USA and Canada. The ADNI was launched in 2003 as a public-private partnership, led by Principal Investigator Michael W. Weiner, MD. The primary goal of ADNI has been to test whether serial magnetic resonance imaging (MRI), PET, other biological markers, and clinical and neuropsychological assessment can be combined to measure the progression of mild cognitive impairment (MCI) and early AD. Further details can be found in Weiner et al.[1].

For this study, 406 cognitively normal (CU), 242 MCI, and 77 AD dementia (ADD) participants who underwent MRI, Aβ-PET, tau-PET, and clinical evaluation within a 6-month window were included in the study.

HABS

HABS is an ongoing, monocentric, longitudinal study of aging conducted at the Massachusetts General Hospital (USA). The HABS study was launched in 2010, funded by the National Institute on Aging. and is led by principal investigators Reisa A. Sperling MD and Keith A. Johnson MD at Massachusetts General Hospital/Harvard Medical School in Boston, MA. Additional details can be found in Dagley et al.[2].

For this study, 167 CU, and 4 AD dementia participants who underwent MRI, Aβ-PET, tau-PET, and clinical evaluation within a 6-month window were included in the study (HABS data release 2.0, obtained April 2021 via habs.mgh.harvard.edu).

AVID-A05

AVID-A05 participants were enrolled in this study from 25 sites. A total of 217 subjects that underwent MRI, Aβ-PET, tau-PET, and clinical evaluation within a 6-month window were included in the study, including 16 young cognitively normal (YCN) and 56 older CU subjects, 97 subjects with MCI, and 48 subjects with clinically-defined possible or probable Alzheimer's disease. YCN subjects were between 20 and 40 years of age, and OCN subjects were >50 years of age. Additional details can be found in Pontecorvo et al.[3].

Image acquisition

ADNI, HABS and AVID-A05 MRI images were acquired using previously published protocols[4–6]. In brief, ADNI FTP-PET images were acquired using dynamic 3D acquisitions of six 5-min frames starting 75 min after the injection of 370 MBq ± 10%. ADNI [^{18}F]florbetapir and [^{18}F]florbetaben scans were acquired using dynamic 3D acquisitions of four 5-min frames 370 MBq ± 10% starting 50 min after injection and 300 MBq ± 10% starting 90 minutes after injection, respectively. For this work, we used images in pre-processing level four as described by ADNI (http://adni.loni.usc.edu/methods/pet-analysis-method/pet-

© 2023 Costoya-Sánchez A et al. *JAMA Neurology*.

analysis), which corresponds to co-registered and averaged images, further reoriented to a standard image matrix and smoothed to 8 mm isotropic resolution.

HABS FTP-PET images were acquired using dynamic 3D acquisitions after the injection of 370 MBq ± 10%. Acquisition varies between four 5-min frames starting at 80 min post-injection and six 5-min frames starting at 75 min post-injection. HABS [^{11}C]PiB PET scans were acquired using dynamic 3D acquisitions of eight 15s, four 1-min and twenty-seven 2-min frames after the injection of 555 MBq. Static PET images were then obtained by realigning and averaging the acquired frames and reslicing the images to the ADNI standard voxel size of 1.5 mm. Afterwards, an isotropic Gaussian filter of FWHM 5 mm was applied to harmonize the images to the ADNI standard resolution of 8 mm.

AVID-A05 FTP-PET images were acquired using dynamic 3D acquisitions of four 5-min frames starting 80 min after the injection of 370 MBq. AVID-A05 [^{18}F]florbetapir scans were acquired using dynamic 3D acquisitions of two 5-min frames. As for HABS PET images, AVID-A05 static PET images were obtained by realigning, averaging the acquired frames and reslicing the images to the ADNI standard voxel size of 1.5 mm. Finally, a scanner-specific isotropic Gaussian filter was applied to harmonize the PET images to the ADNI standard resolution of 8 mm.

Image processing

MRI scans were processed using FreeSurfer (FreeSurfer version 7.1.1, http://surfer.nmr.mgh.harvard.edu) and segmented with SPM (Statistical Parametric Mapping version 12, http://www.fil.ion.ucl.ac.uk/spm). Subject-specific atlases were obtained using the code shared by Baker et al.[7], and its output ROIs were combined to generate the Braak I/II, Braak III/IV and Braak V/VI areas. The definition of the Braak ROIs was identical to the one proposed in Baker et al. Neuroimage. 2017[7], except for the Braak I/II region in which we did not include the hippocampus. We excluded the hippocampus from the Braak I/II ROI for two reasons: 1) the strong influence of spill-in counts from the choroid plexus, as demonstrated in a previous study using Monte Carlo simulation[8] 2) a Braak I/II ROI consisting only of the entorhinal cortex and corrected using Baker's partial volume correction has been shown to be minimally influenced by spill-in counts from the choroid plexus and other off-target regions[8]. The SUIT cerebellar template[9] was transformed to each subject's space using the output of the SPM segmentation to obtain the inferior cerebellum grey matter ROI used as reference region for FTP-PET quantification. The standard Centiloid VOIs were similarly transformed to each subject's space for Centiloid calculation.

PET scans were corregistered to the corresponding MRI using the SPM toolbox. Centiloid calculation was then performed on the corregistered Aβ PET images using the standard Centiloid VOIs following Klunk et al.[10] guidelines. Computed ADNI Centiloid values were used in conjunction with the Aβ PET quantification data provided by ADNI (https://adni.loni.usc.edu/wp-content/themes/freshnews-dev-v2/documents/pet/ADNI%20Centiloids%20Final.pdf) to compute the transformation from the Centiloid values generated by our pipeline to the standard Centiloid units. Additionally, the subject-specific atlas was

© 2023 Costoya-Sánchez A et al. *JAMA Neurology*.

then used for FTP-PET PVC in combination with the PETPVC toolbox[11] using the region-based voxel-wise correction method.

Follow-up Data

Subsets of the study participants underwent follow-up MRI (N=650, 2.00±0.86 years), tau-PET (N=460, 1.83±0.84 years), and Aβ-PET (N=351, 2.36±0.76 years, only ADNI and HABS cohorts). Longitudinal Aβ- and tau-PET scans were only included when they had concurrent MRI scans (6-month window). All participants had baseline cognitive data; longitudinal cognitive assessments were available for n=345 participants over an average mean folloxw-up time of 2.04±1.01 years.

© 2023 Costoya-Sánchez A et al. *JAMA Neurology*.

eFigure 1. MTL tau PET SUVR distribution across groups.

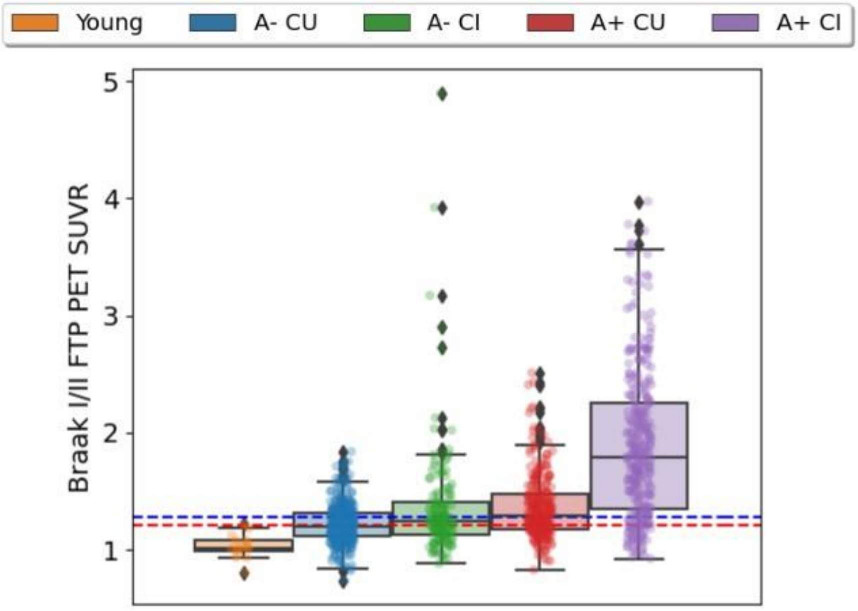

Legend: MTL (Braak I/II ROI) FTP PET SUVR in baseline across all groups (young, A- CU, A- CI, A+ CU, A+ CI individuals). MTL tau positivity cut-points are represented with dotted lines (red: 95th percentile of the MTL SUVR of the young cohort; blue: mean + 2.5 standard deviations of the MTL SUVRs of the young cohort)

© 2023 Costoya-Sánchez A et al. *JAMA Neurology*.

eFigure 2. MTL tau-PET positivity across age and sex.

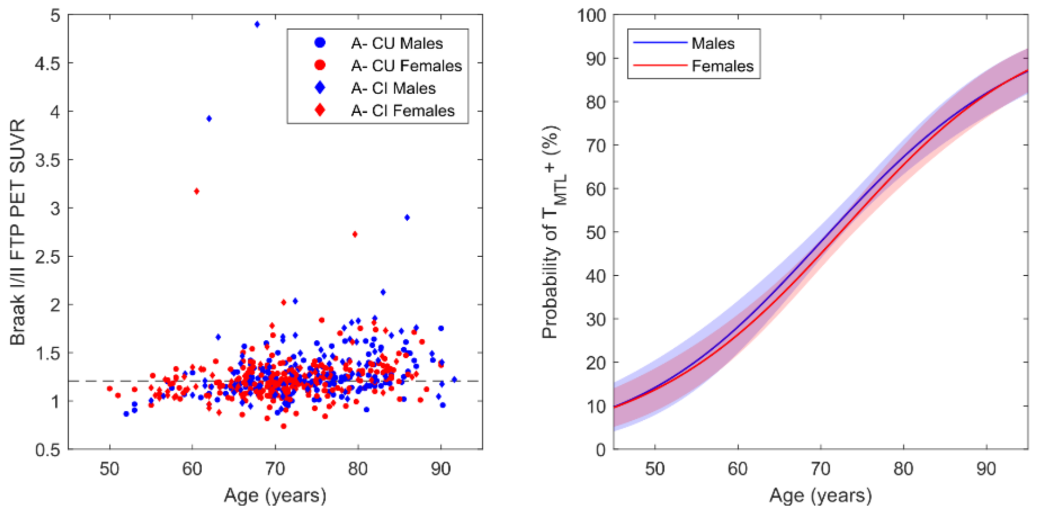

Legend: Left: Baseline MTL (Braak I/II ROI) FPT PET SUVR relation with age. Dotted blue line indicates MTL tau positivity cutpoint. Right: Probability of MTL tau positivity for each sex.

© 2023 Costoya-Sánchez A et al. *JAMA Neurology*.

eFigure 3. Baseline levels and longitudinal change rates of ROI-wise FTP-PET SUVR values.

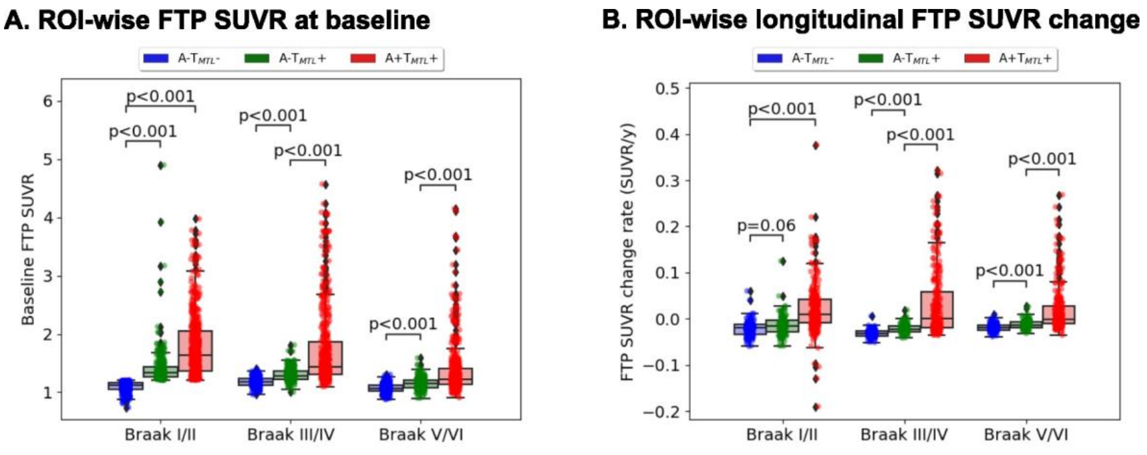

Legend: A) Cross-sectional distribution of ROI-wise FTP-PET SUVR across groups compared using GLM models and the A-T- group as the reference (Braak I/II: A-T_{MTL}+, d=1.09, p<0.001; A+T_{MTL}+, d=1.40, p<0.001; Braak III/IV: A-T_{MTL}+, d=0.99, p<0.001; A+T_{MTL}+, d=1.03, p<0.001; Braak V/VI: A-T_{MTL}+, d=0.72; A+T_{MTL}+, d=0.93). B) Longitudinal progression of ROI-wise FTP-PET SUVR across groups, obtained using LME models and compared with GLMs using the A-T- group as the reference (Braak I/II: A-T_{MTL}+, d=0.24; A+T_{MTL}+, d=0.80; Braak III/IV: A-T_{MTL}+, d=0.89; A+T_{MTL}+, d=1.17; Braak V/VI: A-T_{MTL}+, d=0.67; A+T_{MTL}+, d=1.11).

© 2023 Costoya-Sánchez A et al. *JAMA Neurology*.

eFigure 4. Longitudinal change from baseline in Centiloids across groups.

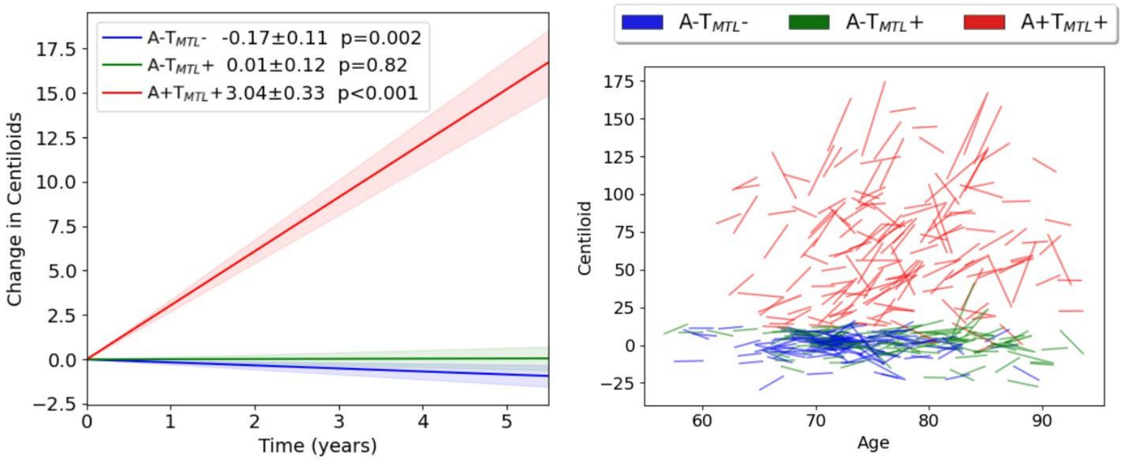

Legend: Left: Average change from baseline in Centiloids and 95% confidence interval for the A-T_{MTL}-, A-T_{MTL}+ and A+T_{MTL}+ groups. Reported values in the legend represent annual change in Centiloid values, together with standard errors. Reported p-values correspond to a one-sample t-test testing the null hypothesis of no change over time. Right: Spaghetti plot of Centiloid values across all subjects for each of their age values, grouped by A- T_{MTL}-, A-T_{MTL}+ and A+T_{MTL}+

© 2023 Costoya-Sánchez A et al. *JAMA Neurology*.

eFigure 5. Association of baseline ERC FTP SUVR with baseline cortical thickness in A- individuals.

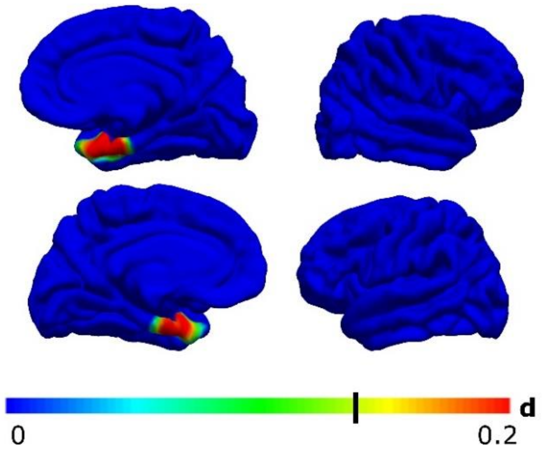

Legend: Association of baseline ERC FTP SUVR with vertex-wise cortical thickness in the pooled group of A-T_{MTL}- and A-T_{MTL}+ individuals, represented as Cohen's d. The black line in the colorbar indicates the statistical significance threshold using a FDR α=0.05 (d=0.13).

© 2023 Costoya-Sánchez A et al. *JAMA Neurology*.

eFigure 6. Longitudinal change from baseline in group-wise ADAS-Cog 11 scores in CI individuals.

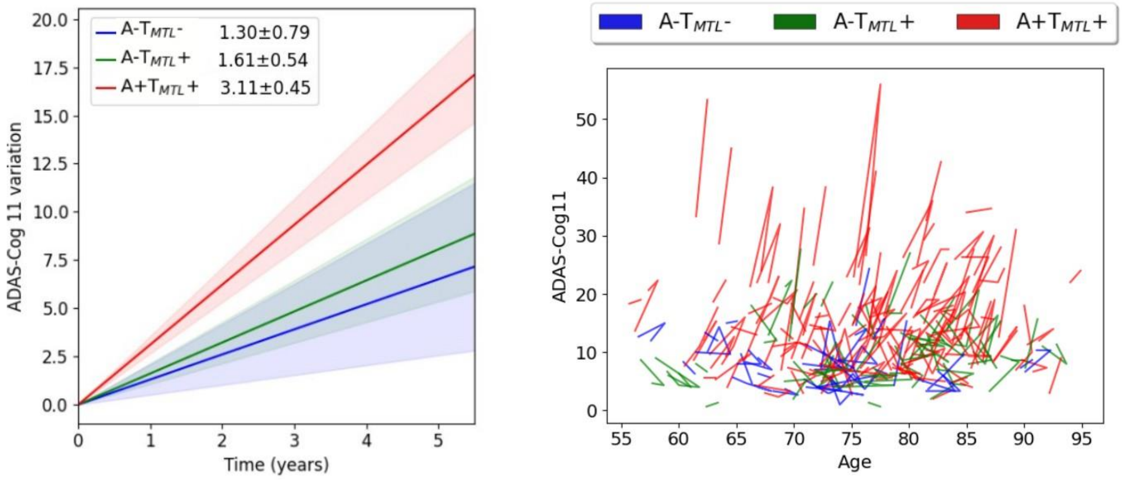

Legend: Left: Average change from baseline in ADAS-Cog 11 and 95% confidence interval for the A-T_{MTL}-, A-T_{MTL}+ and A+T_{MTL}+ CI groups. Reported values in the legend represent annual change in ADAS-Cog 11, together with standard errors. Reported p-values correspond to a one-sample t-test testing the null hypothesis of no change over time. Right: Spaghetti plot of ADAS-Cog 11 values across all CI subjects for each of their age values, grouped by A- T_{MTL}-, A-T_{MTL}+ and A+T_{MTL}+

© 2023 Costoya-Sánchez A et al. *JAMA Neurology*.

eFigure 7. Cross-sectional and longitudinal tau- and Aβ-PET accumulation, using an Aβ-PET positivity cutpoint of 24 Centiloids.

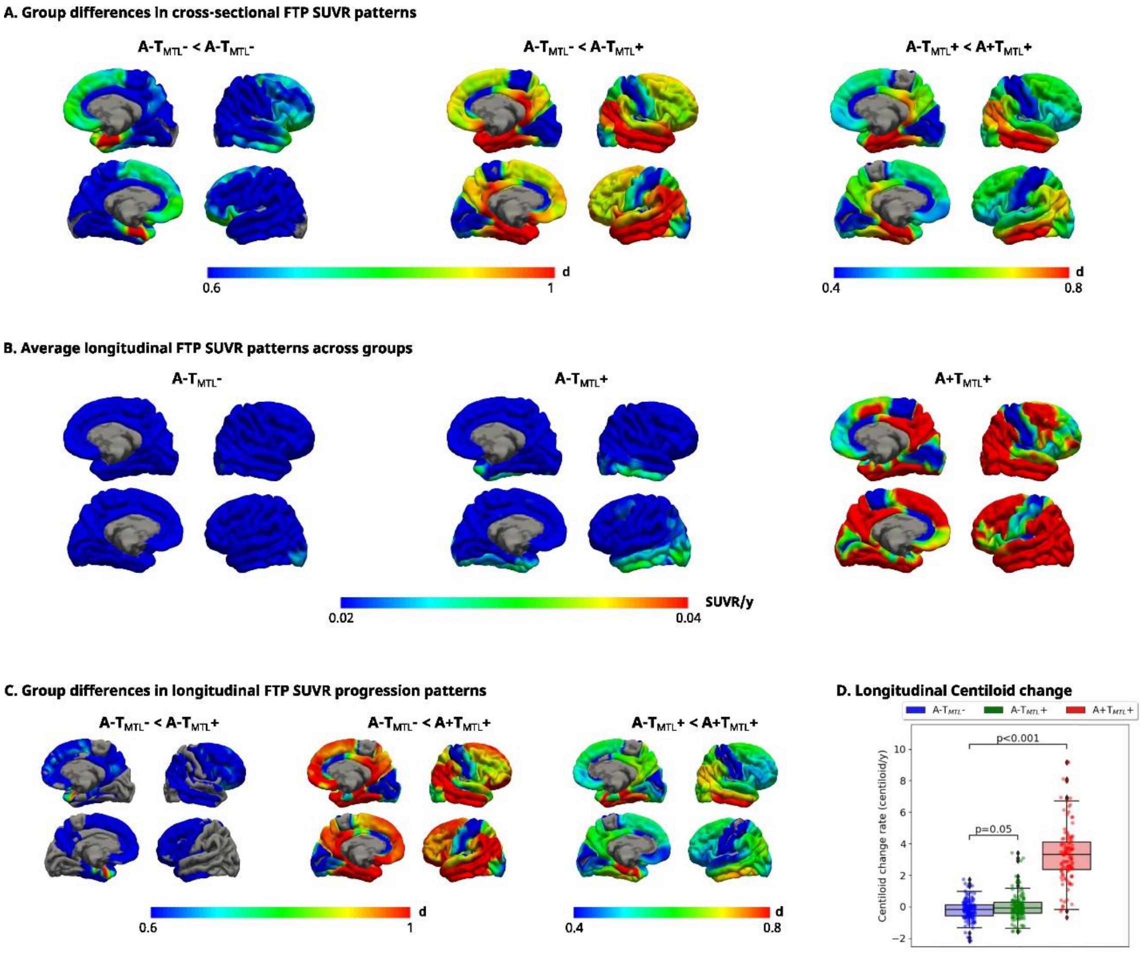

Legend: Group composition: 311 A-T_{MTL}-, 336 A-T_{MTL}+ and 383 A+T_{MTL}+. A) Vertex-wise group differences in cross-sectional FTP SUVR in A-T_{MTL}+ and A+T_{MTL}+ individuals compared to the A-T_{MTL}- control group, expressed as Cohen's d. B) Average longitudinal FTP SUVR in A-T_{MTL}-, A-T_{MTL}+, and A+T_{MTL}+ individuals, represented as vertex-wise rates of change. C) Vertex-wise group differences in longitudinal FTP SUVR in A-T_{MTL}+ and A+T_{MTL}+ individuals compared to the A-T_{MTL}- control group, expressed as Cohen's d. D) Longitudinal Centiloid change in A-T_{MTL}-, A-T_{MTL}+, and A+T_{MTL}+ individuals.

© 2023 Costoya-Sánchez A et al. *JAMA Neurology*.

eFigure 8. Baseline and longitudinal CSF Aβ and tau biomarker levels, using an Aβ-PET positivity cut-point of 24 Centiloids.

A. CSF biomarker distribution at baseline

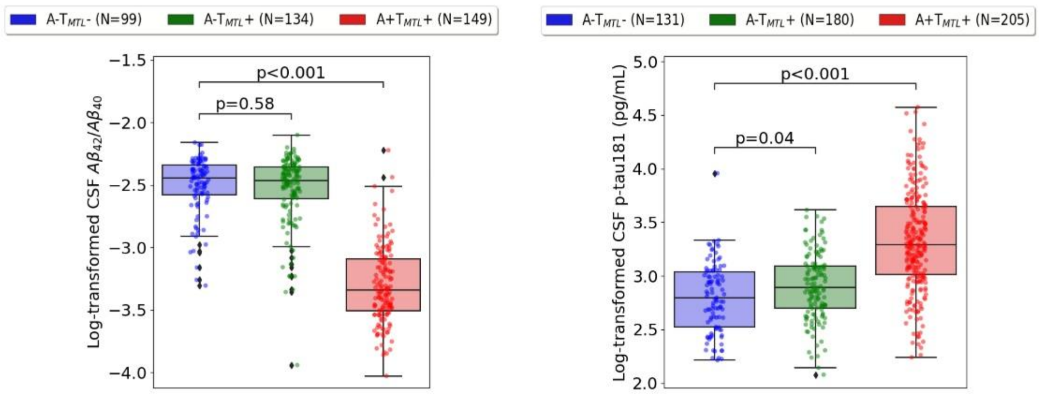

B. Longitudinal CSF biomarker change

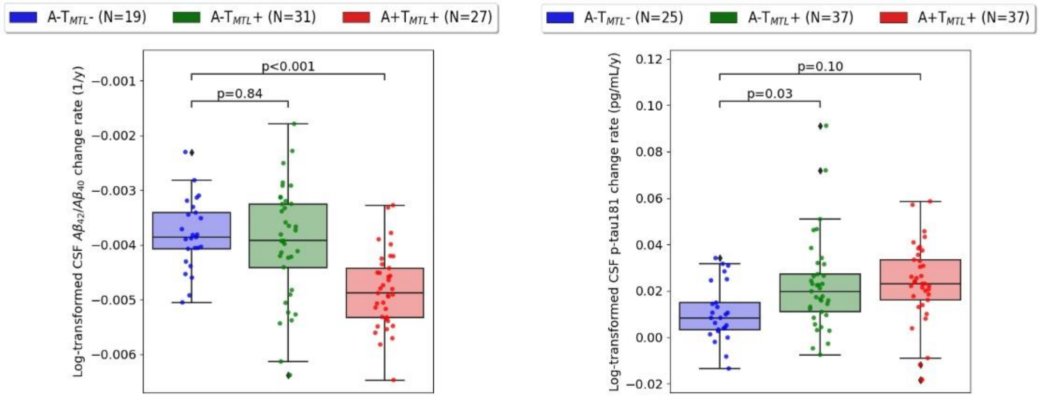

Legend: Group composition: 311 A-T$_{MTL}$-, 336 A-T$_{MTL}$+ and 383 A+T$_{MTL}$+. A) Baseline CSF A$\beta_{42/40}$ and p-tau$_{181}$ levels across groups. Biomarker levels were statistically compared using GLM models. B) Longitudinal change of CSF A$\beta_{42/40}$ and p-tau$_{181}$ metrics across groups, obtained using LME models and statistically compared with GLMs.

© 2023 Costoya-Sánchez A et al. *JAMA Neurology*.

eFigure 9. Baseline and longitudinal cortical thickness, using an Aβ-PET positivity cut-point of 24 Centiloids.

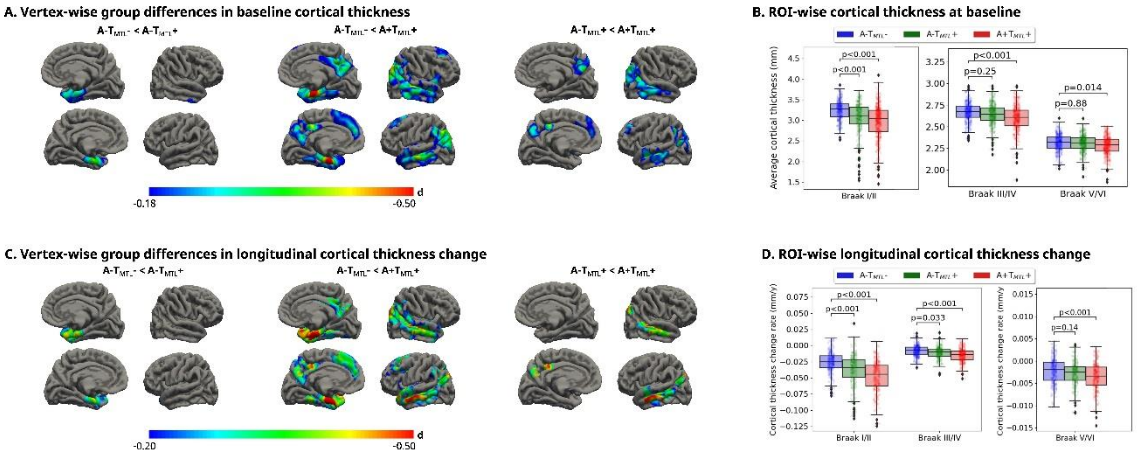

Legend: Group composition: 311 A-T_{MTL}-, 336 A-T_{MTL}+ and 383 A+T_{MTL}+. A) Vertex-wise group differences in baseline cortical thickness in A-T_{MTL}+ and A+T_{MTL}+ individuals compared to the A-T_{MTL}- control group, expressed as Cohen's d. B) Regional group differences in baseline cortical thickness in A-T_{MTL}+ and A+T_{MTL}+ individuals compared to the A-T_{MTL}- control group using GLM models (ns=non-significant, *=*p*<0.05). C) Vertex-wise group differences in longitudinal cortical thickness progression patterns in A-T_{MTL}+ and A+T_{MTL}+ individuals compared to the A-T_{MTL}- control group, measured as Cohen's d. D) Regional group differences in longitudinal cortical thickness progression patterns in A-T_{MTL}+ and A+T_{MTL}+ individuals compared to the A-T_{MTL}- control group using GLM models (ns=non-significant, *=*p*<0.05).

© 2023 Costoya-Sánchez A et al. *JAMA Neurology*.

eFigure 10. Baseline and longitudinal cognitive performance, using an Aβ-PET positivity cut-point of 24 Centiloids.

A. Cognitive performance at baseline

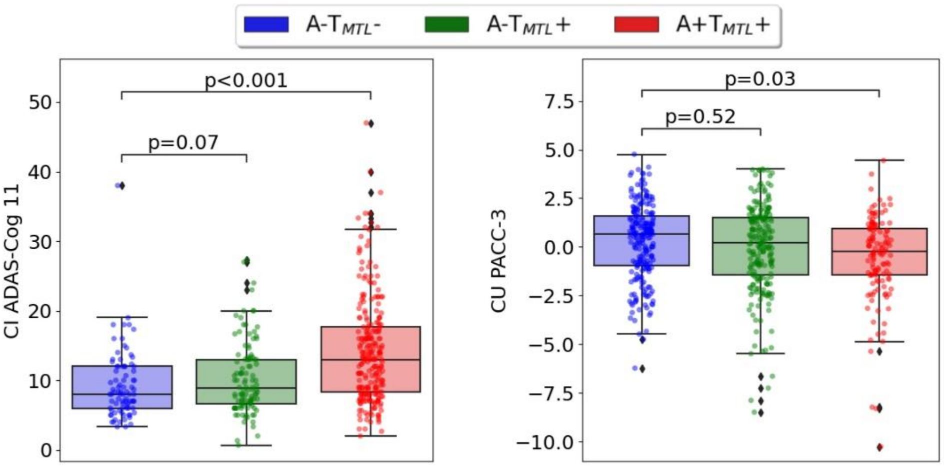

B. Longitudinal cognitive performance change

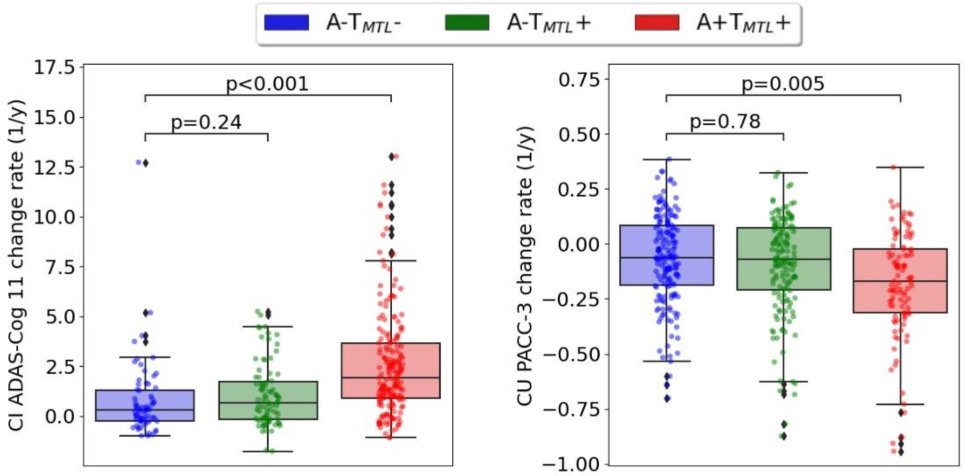

Legend: Group composition: 311 A-T_{MTL}-, 336 A-T_{MTL}+ and 383 A+T_{MTL}+. A) Baseline ADAS-Cog 11 scores in CI individuals and PACC-3 scores in CU individuals across A/T_{MTL} groups, compared using GLMs. B) Longitudinal changes in ADAS-Cog 11 scores in CI individuals and PACC-3 scores in CU individuals across A/T_{MTL} groups, obtained with LME models and compared using GLMs.

© 2023 Costoya-Sánchez A et al. *JAMA Neurology*.

eFigure 11. Cross-sectional and longitudinal tau- and Aβ-PET accumulation, using a tau-PET positivity SUVR cut-point of 1.27.

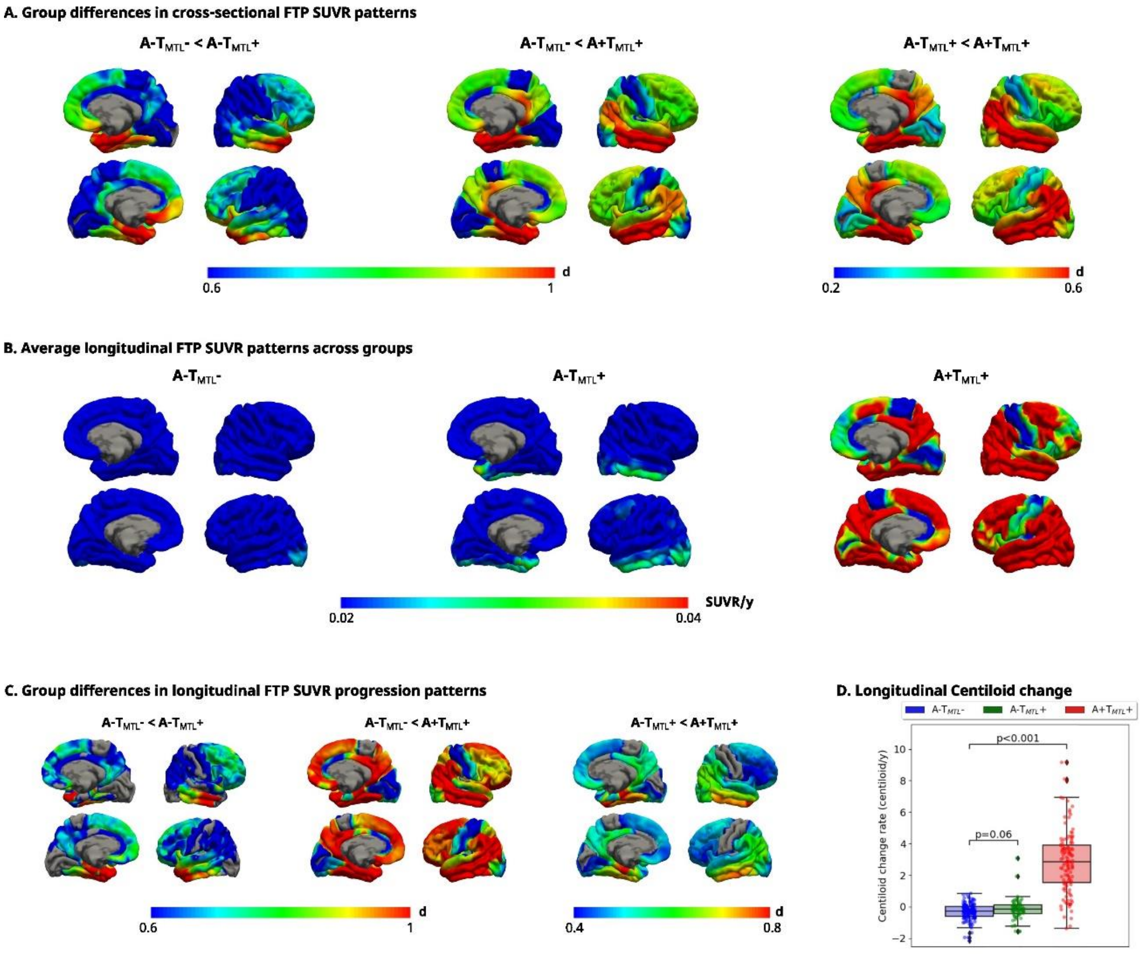

Legend: Group composition: 335 A-T_{MTL}-, 179 A-T_{MTL}+ and 396 A+T_{MTL}+. A) Vertex-wise group differences in cross-sectional FTP SUVR in A-T_{MTL}+ and A+T_{MTL}+ individuals compared to the A-T_{MTL}- control group, expressed as Cohen's d. B) Average longitudinal FTP SUVR in A-T_{MTL}-, A-T_{MTL}+, and A+T_{MTL}+ individuals, represented as vertex-wise rates of changes. C) Vertex-wise group differences in longitudinal FTP SUVR in A-T_{MTL}+ and A+T_{MTL}+ individuals compared to the A-T_{MTL}- control group, expressed as Cohen's d. D) Longitudinal Centiloid change in A-T_{MTL}-, A-T_{MTL}+, and A+T_{MTL}+ individuals.

© 2023 Costoya-Sánchez A et al. *JAMA Neurology*.

eFigure 12. Baseline and longitudinal CSF Aβ and tau biomarker levels, using a tau-PET positivity SUVR cut-point of 1.27.

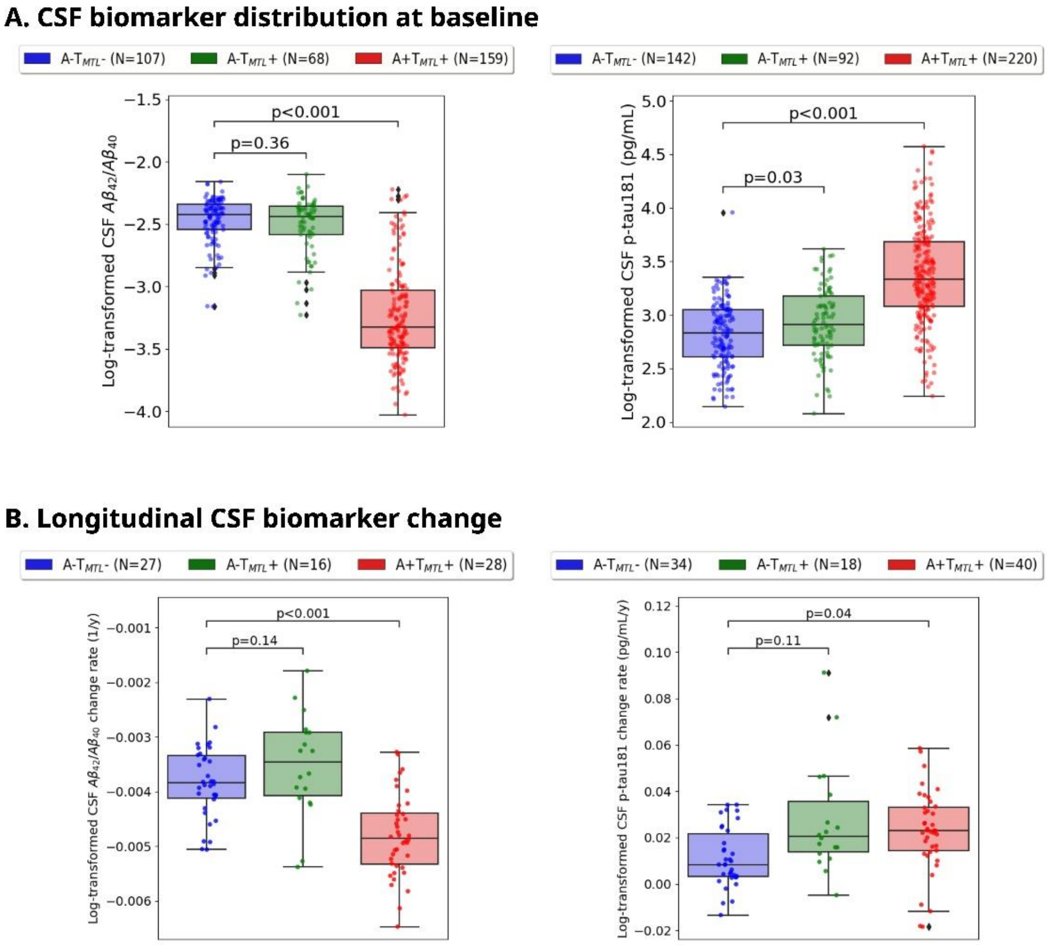

Legend: Group composition: 335 A-T_{MTL}-, 179 A-T_{MTL}+ and 396 A+T_{MTL}+. A) Baseline CSF $A\beta_{42/40}$ and p-tau_{181} levels across groups. Biomarker levels were statistically compared using GLM models. B) Longitudinal change of CSF $A\beta_{42/40}$ and p-tau_{181} metrics across groups, obtained using LME models and statistically compared with GLMs.

© 2023 Costoya-Sánchez A et al. *JAMA Neurology*.

eFigure 13. Baseline and longitudinal cortical thickness, using a tau-PET positivity SUVR cut-point of 1.27.

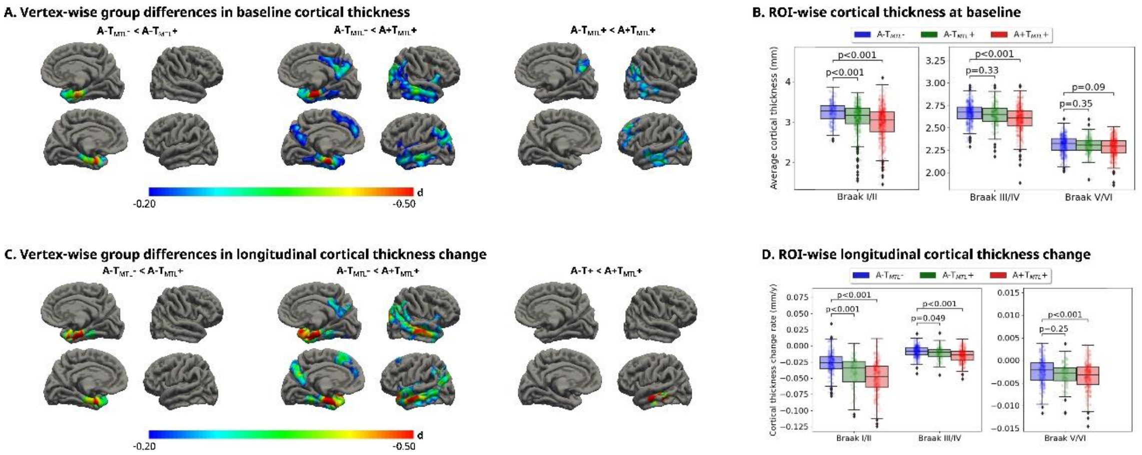

Legend: Group composition: 335 A-T_{MTL}-, 179 A-T_{MTL}+ and 396 A+T_{MTL}+. A) Vertex-wise group differences in baseline cortical thickness in A-T_{MTL}+ and A+T_{MTL}+ individuals compared to the A-T_{MTL}- control group, measured as Cohen's d. B) Regional group differences in baseline cortical thickness in A-T_{MTL}+ and A+T_{MTL}+ individuals compared to the A-T_{MTL}- control group using GLM models (ns=non-significant, *=p<0.05). C) Vertex-wise group differences in longitudinal cortical thickness change in A-T_{MTL}+ and A+T_{MTL}+ individuals compared to the A-T_{MTL}- control group, measured as Cohen's d. D) Regional group differences in longitudinal cortical thickness change in A-T_{MTL}+ and A+T_{MTL}+ individuals compared to the A-T_{MTL}- control group using GLM models (ns=non-significant, *=p<0.05).

eFigure 14. Baseline and longitudinal cognitive performance, using a tau-PET positivity SUVR cut-point of 1.27.

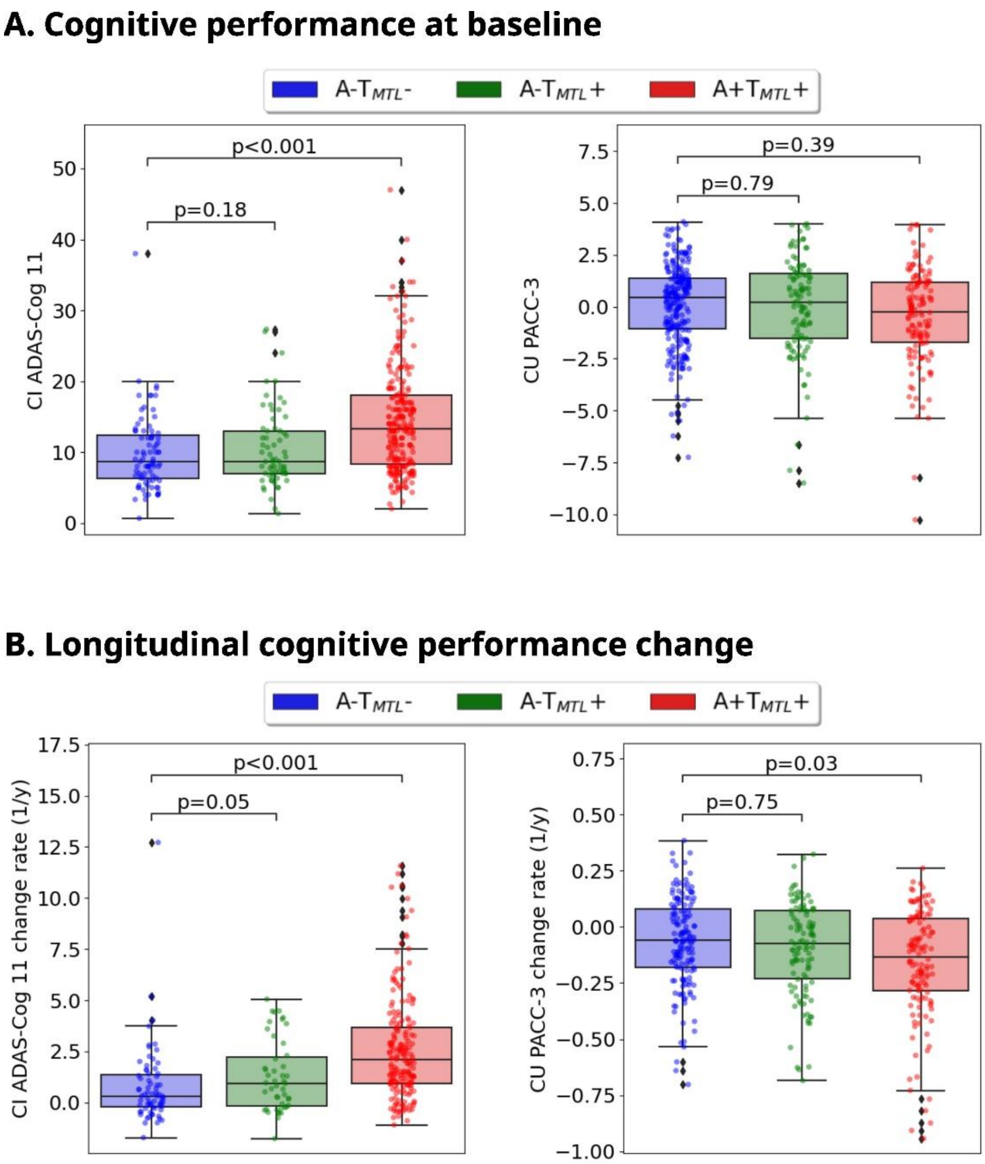

Legend: Group composition: 335 A-T_{MTL}-, 179 A-T_{MTL}+ and 396 A+T_{MTL}+. A) Baseline ADAS-Cog 11 scores in CI individuals and PACC-3 scores in CU individuals across A/T_{MTL} groups, compared using GLMs. B) Longitudinal changes in ADAS-Cog 11 scores in CI individuals and PACC-3 scores in CU individuals across A/T_{MTL} groups, obtained with LME models and compared using GLMs.

© 2023 Costoya-Sánchez A et al. *JAMA Neurology*.

eFigure 15. Cross-sectional and longitudinal tau- and Aβ-PET accumulation, using an entorhinal cortex & amygdala ROI.

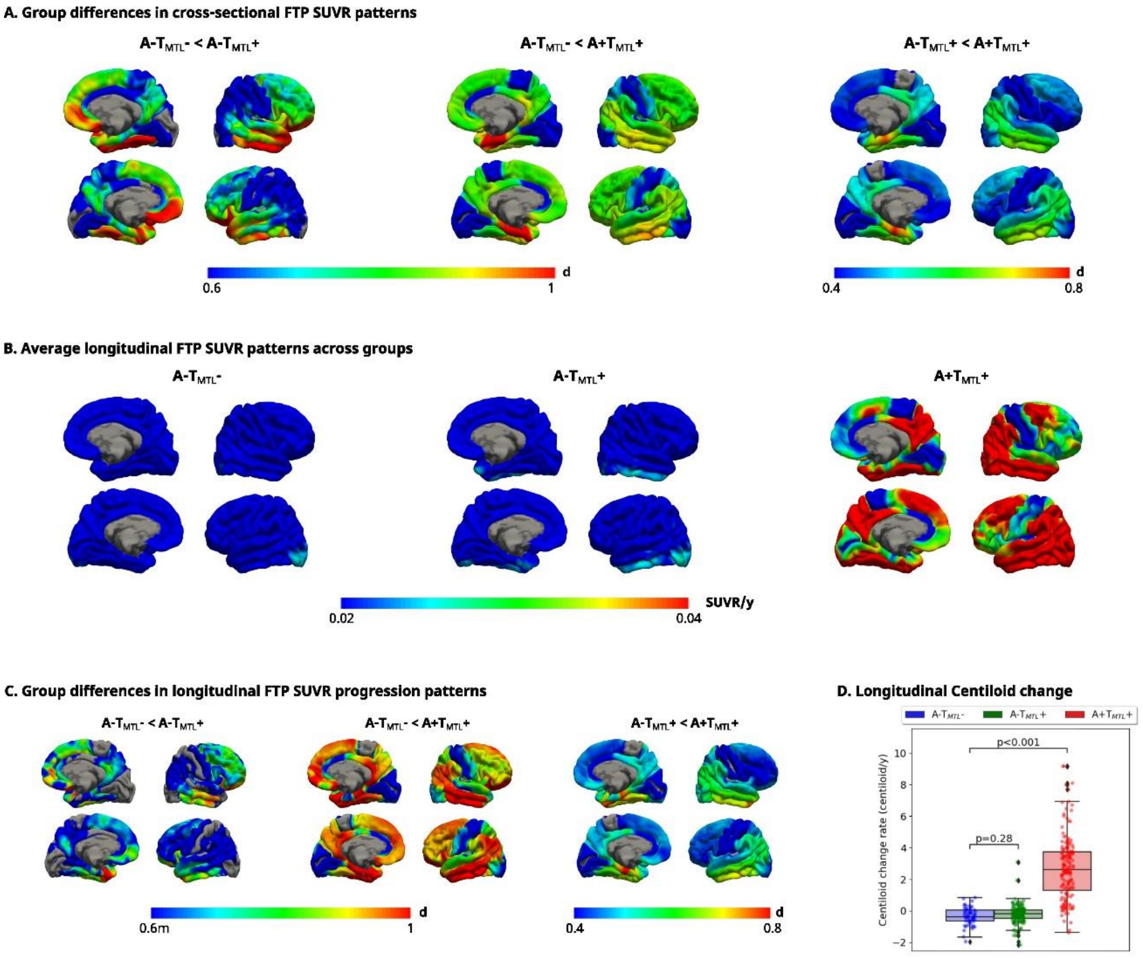

Legend: Group composition: 170 A-T_{MTL}-, 344 A-T_{MTL}+ and 512 A+T_{MTL}+. A) Vertex-wise group differences in cross-sectional FTP SUVR patterns in A-T_{MTL}+ and A+T_{MTL}+ individuals compared to the control group, expressed as Cohen's d. B) Average longitudinal FTP SUVR patterns in A-T_{MTL}-, A-T_{MTL}+, and A+T_{MTL}+ individuals, represented as vertex-wise rates of changes. C) Vertex-wise group differences in longitudinal FTP SUVR in A-T_{MTL}+ and A+T_{MTL}+ individuals compared to the A-T_{MTL}- control group, expressed as Cohen's d. D) Longitudinal centiloid change in A-T_{MTL}-, A-T_{MTL}+, and A+T_{MTL}+ individuals.

© 2023 Costoya-Sánchez A et al. *JAMA Neurology*.

eReferences

1. Weiner, M. W. *et al.* The Alzheimer's Disease Neuroimaging Initiative 3: Continued innovation for clinical trial improvement. *Alzheimer's & Dementia* **13**, 561–571 (2017).
2. Dagley, A. *et al.* Harvard Aging Brain Study: Dataset and accessibility. *NeuroImage* **144**, 255–258 (2017).
3. Pontecorvo, M. J. *et al.* Relationships between flortaucipir PET tau binding and amyloid burden, clinical diagnosis, age and cognition. *Brain* aww334 (2017) doi:10.1093/brain/aww334.
4. Pontecorvo, M. J. *et al.* A multicentre longitudinal study of flortaucipir (18F) in normal ageing, mild cognitive impairment and Alzheimer's disease dementia. *Brain* **142**, 1723–1735 (2019).
5. Hanseeuw, B. J. *et al.* Association of Amyloid and Tau With Cognition in Preclinical Alzheimer Disease: A Longitudinal Study. *JAMA Neurol* **76**, 915 (2019).
6. Jagust, W. J. *et al.* The Alzheimer's Disease Neuroimaging Initiative positron emission tomography core. *Alzheimer's & Dementia* **6**, 221–229 (2010).
7. Baker, S. L., Maass, A. & Jagust, W. J. Considerations and code for partial volume correcting [18 F]-AV-1451 tau PET data. *Data in Brief* **15**, 648–657 (2017).
8. López-González, F. J. *et al.* Impact of spill-in counts from off-target regions on [18F]Flortaucipir PET quantification. *NeuroImage* **259**, 119396 (2022).
9. Diedrichsen, J. A spatially unbiased atlas template of the human cerebellum. *NeuroImage* **33**, 127–138 (2006).
10. Klunk, W. E. *et al.* The Centiloid Project: Standardizing quantitative amyloid plaque estimation by PET. *Alzheimer's & Dementia* **11**, 1 (2015).
11. Thomas, B. A. *et al.* PETPVC: a toolbox for performing partial volume correction techniques in positron emission tomography. *Phys. Med. Biol.* **61**, 7975–7993 (2016).

© 2023 Costoya-Sánchez A et al. *JAMA Neurology*.
