# Supplementary material for: Increased Medial Temporal Tau Positron Emission Tomography Uptake in the Absence of Amyloid-β Positivity
Source: JAMA Neurol. 2023 Aug 14;80(10):1051–61. doi: 10.1001/jamaneurol.2023.2560 (PMC10425864; doi:10.1001/jamaneurol.2023.2560)
Supplement: Supplement 2. — Nonauthor Collaborators. The Alzheimer’s Disease Neuroimaging Initiative and the Harvard Aging Brain Study [file jamaneurol-e232560-s002.pdf]

Supplemental Online Content: Nonauthor Collaborators
*First name, last name, and suffix (if applicable) are required and will appear in PubMed.

*Group Name(s): Alzheimer's Disease Neuroimaging Initiative and the Harvard Aging Brain Study							
***First Name and Middle Initial(s)**	***Last Name**	***Suffix (eg, Jr, III)**	Academic Degrees	Institution	Location (city, state/province, country)	Role or Contribution, eg, chair, principal investigator	Group (if more than 1 Group listed in the byline) and/or Subgroup (eg, Steering Committee)
Michael W	Weiner		MD	UCSF			ADNI
Paul	Aisen		MD	USC			ADNI
Ronald	Petersen		MD PhD	Mayo Clinic, Rochester			ADNI
Clifford R	Jack	Jr	MD	Mayo Clinic, Rochester			ADNI
William	Jagust		MD	UC Berkeley			ADNI
John Q	Trojanowki		MD PhD	UPenn			ADNI
Arthur W	Toga		PhD	USC			ADNI
Laurel	Beckett		PhD	UC Davis			ADNI
Robert C	Green		MD MPH	Brigham and Women's Hospital/Harvard Medical School			ADNI
Andrew J	Saykin		PsyD	Indiana University			ADNI
John C	Morris		MD	Washington University St. Louis			ADNI
Richard J	Perrin		MD PhD	Washington University St. Louis			ADNI
Leslie M	Shaw		PhD	UPenn			ADNI
Zaven	Khachaturian		PhD	Prevent Alzheimer's Disease 2020			ADNI
Maria	Carrillo		PhD	Alzheimer's Association			ADNI
William	Potter		PhD	NIMH			ADNI
Lisa	Barnes		PhD	Rush University			ADNI
Marie	Bernard		MD	NIA			ADNI
Hector	Gonzalez			USSD			ADNI
Carole	Ho			Denali Therapeutics			ADNI
John K	Hsiao		MD	NIH			ADNI
Jonathan	Jackson		PhD	MGH			ADNI
Eliezer	Masliah		MD	NIA			ADNI
Donna	Masterman		MD	Biogen			ADNI
Ozioma	Okonkwo		PhD	University of Wisconsin Madison			ADNI
Laurie	Ryan		PhD	NIA			ADNI
Nina	Silverberg		PhD	NIA			ADNI
Adam	Fleisher		MD	Eli Lilly			ADNI

Supplemental Online Content: Nonauthor Collaborators
*First name, last name, and suffix (if applicable) are required and will appear in PubMed.

***First Name and Middle Initial(s)**	***Last Name**	***Suffix (eg, Jr, III)**	Academic Degrees	Institution	Location (city, state/province, country)	Role or Contribution, eg, chair, principal investigator	Group (if more than 1 Group listed in the byline) and/or Subgroup (eg, Steering Committee)
Diana T	Sacrey			NCIRE/The Veterans Health Research Institute			ADNI
Juliet	Fockler			UCSF			ADNI
Cat	Conti		BA	NCIRE/The Veterans Health Research Institute			ADNI
Dallas	Veitch		PhD	NCIRE/The Veterans Health Research Institute			ADNI
John	Neuhaus		PhD	UCSF			ADNI
Chengshi	Jin		PhD	UCSF			ADNI
Rachel	Nosheny		PhD	UCSF			ADNI
Mariam	Ashford		PhD	NCIRE/The Veterans Health Research Institute			ADNI
Derek	Flenniken			NCIRE/The Veterans Health Research Institute			ADNI
Adrienne	Kormos			NCIRE/The Veterans Health Research Institute			ADNI
Tom	Montine		MD PhD	NCIRE/The Veterans Health Research Institute			ADNI
Cat	Conti		BA	NCIRE/The Veterans Health Research Institute			ADNI
Michael	Rafii		MD PhD	USC			ADNI
Rema	Raman		PhD	USC			ADNI
Gustavo	Jimenez		MBS	USC			ADNI
Michael	Donohue		PhD	USC			ADNI
Devon	Gessert		BS	USC			ADNI
Jennifer	Salazar		MBS	USC			ADNI
Caileigh	Zimmerman		MS	USC			ADNI
Yuliana	Cabrera		BS	USC			ADNI
Sarah	Walter		MSc	USC			ADNI
Garrett	Miller		MS	USC			ADNI

Supplemental Online Content: Nonauthor Collaborators
*First name, last name, and suffix (if applicable) are required and will appear in PubMed.

***First Name and Middle Initial(s)**	***Last Name**	***Suffix (eg, Jr, III)**	Academic Degrees	Institution	Location (city, state/province, country)	Role or Contribution, eg, chair, principal investigator	Group (if more than 1 Group listed in the byline) and/or Subgroup (eg, Steering Committee)
Godfrey	Coker		MBA MPH	USC			ADNI
Taylor	Clanton		MPH	USC			ADNI
Lindsey	Hergesheimer		BS	USC			ADNI
Stephanie	Smith		BS	USC			ADNI
Olusegun	Adegoke		MSc	USC			ADNI
Payam	Mahboubi		MPH	USC			ADNI
Shelley	Moore		BA	USC			ADNI
Jeremy	Pizzola		BA	USC			ADNI
Elizabeth	Shaffer		BS	UC Davis			ADNI
Laurel	Beckett		PhD	UC Davis			ADNI
Danielle	Harvey		PhD	Mayo Clinic			ADNI
Arvin	Forghanian-Arani		PhD	Mayo Clinic			ADNI
Bret	Borowski		RTR	Mayo Clinic			ADNI
Chad	Ward			Mayo Clinic			ADNI
Christopher	Schwarz		PhD	Mayo Clinic			ADNI
David	Jones		MD	Mayo Clinic			ADNI
Jeff	Gunter		PhD	Mayo Clinic			ADNI
Kejal	Kantarci		MD	Mayo Clinic			ADNI
Matthew	Senjem		MS	Mayo Clinic			ADNI
Prashanthi	Vemuri		PhD	Mayo Clinic			ADNI
Robert	Reid		PhD	Mayo Clinic			ADNI
Nick C	Fox		MD	University College London			ADNI
Ian	Malone		PhD	University College London			ADNI
Paul	Thompson		PhD	USC School of Medicine			ADNI
Sophia I	Thomopoulos		BS	USC School of Medicine			ADNI
Talia M	Nir		PhD	USC School of Medicine			ADNI
Neda	Jahanshad		PhD	USC School of Medicine			ADNI
Charles	DeCarli		MD	UC Davis			ADNI
Alexander	Knaack		MS	UC Davis			ADNI
Evan	Fletcher		PhD	UC Davis			ADNI

Supplemental Online Content: Nonauthor Collaborators
*First name, last name, and suffix (if applicable) are required and will appear in PubMed.

***First Name and Middle Initial(s)**	***Last Name**	***Suffix (eg, Jr, III)**	Academic Degrees	Institution	Location (city, state/province, country)	Role or Contribution, eg, chair, principal investigator	Group (if more than 1 Group listed in the byline) and/or Subgroup (eg, Steering Committee)
Duygu	Tosun-Turgut		PhD	UCSF			ADNI
Stephanie R	Chen		BA	NCIRE/The Veterans Health Research Institute			ADNI
Mark	Choe		BS	NCIRE/The Veterans Health Research Institute			ADNI
Karen	Crawford			USC School of Medicine			ADNI
Paul A	Yuschkevich		PhD	UPenn			ADNI
Sandhitsu	Das		PhD	UPenn			ADNI
Robert A	Koeppe		PhD	University of Michigan			ADNI
Eric M	Reiman		MD	Banner Alzheimer's Institute			ADNI
Kewei	Chen		PhD	Banner Alzheimer's Institute			ADNI
Chet	Mathis		MD	University of Pittsburgh			ADNI
Susan	Landau		PhD	UC Berkeley			ADNI
Nigel J	Cairns		PhD FRCPath	Washington University St. Louis - Past Investigator			ADNI
Erin	Householder		MS	Washington University St. Louis			ADNI
Erin	Franklin		MS	Washington University St. Louis			ADNI
Haley	Bernhardt		BA	Washington University St. Louis			ADNI
Lisa	Taylor-Reinwald		BA	Washington University St. Louis			ADNI
Magdalena	Korecka		PhD	UPenn			ADNI
Michal	Figurski		PhD	UPenn			ADNI
Scott	Neu		PhD	USC			ADNI
Kwangsik	Nho		PhD	Indiana University			ADNI
Shannon L	Risacher		PhD	Indiana University			ADNI
Liana G	Apostolova		MD	Indiana University			ADNI
Li	Shen		PhD	UPenn			ADNI
Tatiana M	Foroud		PhD	NCRAD/Indiana University School of Medicine			ADNI

*First name, last name, and suffix (if applicable) are required and will appear in PubMed.

***First Name and Middle Initial(s)**	***Last Name**	***Suffix (eg, Jr, III)**	Academic Degrees	Institution	Location (city, state/province, country)	Role or Contribution, eg, chair, principal investigator	Group (if more than 1 Group listed in the byline) and/or Subgroup (eg, Steering Committee)
Kelly	Nudelman		PhD	NCRAD/Indiana University School of Medicine			ADNI
Kelley	Faber		MS	NCRAD/Indiana University School of Medicine			ADNI
Kristi	Wilmes		MS	NCRAD/Indiana University School of Medicine			ADNI
Leon	Thal		MD	UCSD			ADNI
Keith A	Johnson		MD	MGH/Harvard Medical School			HABS
Reisa A	Sperling		MD	MGH/Harvard Medical School			HABS
Dorene	Rentz						HABS
Rebecca	E. Amariglio						HABS
Deborah	Blacker						HABS
Rachel	Buckley						HABS
Jasmeer P.	Chhatwal						HABS
Brad	Dickerson						HABS
Nancy	Donovan						HABS
Michelle	Farrell						HABS
Geoffroy	Gagliardi						HABS
Jennifer	Gatchel						HABS
Edmarie	Guzman-Velez						HABS
Heidi	Jacobs						HABS
Roos	Jutten						HABS
Cristina	Lois Gomez						HABS
Gad	Marshall						HABS
Kate	Oaoo						HABS
Enmanuelle	Pardilla-Delgado						HABS

Supplemental Online Content: Nonauthor Collaborators
*First name, last name, and suffix (if applicable) are required and will appear in PubMed.

***First Name and Middle Initial(s)**	***Last Name**	***Suffix (eg, Jr, III)**	Academic Degrees	Institution	Location (city, state/province, country)	Role or Contribution, eg, chair, principal investigator	Group (if more than 1 Group listed in the byline) and/or Subgroup (eg, Steering Committee)
Juliet	Price						HABS
Prokopis	Prokopiou						HABS
Yakeel	Quiroz						HABS
Gretchen	Reynolds						HABS
Aaron	Schultz						HABS
Stephanie	Schultz						HABS
Jorge	Sepulcre						HABS
Irina	Skylar-Scott						HABS
Patrizia	Vannini						HABS
Clara	Vila-Castelar						HABS
Hyun-Sik	Yang						HABS
