# Supplementary material for: Increased Medial Temporal Tau Positron Emission Tomography Uptake in the Absence of Amyloid-β Positivity
Source: JAMA Neurol. 2023 Aug 14;80(10):1051–61. doi: 10.1001/jamaneurol.2023.2560 (PMC10425864; doi:10.1001/jamaneurol.2023.2560)
Supplement: Supplement 3. — Data Sharing Statement [file jamaneurol-e232560-s003.pdf]

Data Sharing Statement

Costoya-Sánchez. Increased Medial Temporal Tau Positron Emission Tomography Uptake in the Absence of Amyloid-β Positivity. *JAMA Neurol*. Published August 14, 2023. doi:10.1001/jamaneurol.2023.2560

Data
Data available: No

Additional Information
Explanation for why data not available: Data will be available upon request
